# Analyzing Thalamocortical Tract-Tracing Experiments in a Common Reference Space

**DOI:** 10.1007/s12021-023-09644-4

**Published:** 2023-10-21

**Authors:** Nestor Timonidis, Mario Rubio-Teves, Carmen Alonso-Martínez, Rembrandt Bakker, María García-Amado, Paul Tiesinga, Francisco Clascá

**Affiliations:** 1 Neuroinformatics Department, Donders Centre for Neuroscience, Radboud University Nijmegen, Heyendaalseweg 135, 6525 AJ Nijmegen, The Netherlands; 2 Department of Anatomy and Neuroscience, School of Medicine, Autónoma de Madrid University, C. Arzobispo Morcillo 4, 28029 Madrid, Spain; 3 Inst. of Neuroscience and Medicine (INM-6) and Inst. for Advanced Simulation (IAS-6) and JARA BRAIN Inst. I, Jülich Research Centre, Wilhelm-Johnen-Strasse, 52425 Jülich, Germany

**Keywords:** Common coordinate framework, Spatial registration, Somatosensory system, Thalamus, Cortex, Topography, Somatotopy, Tract-tracing, Connectomics, VPM

## Abstract

Current mesoscale connectivity atlases provide limited information about the organization of thalamocortical projections in the mouse brain. Labeling the projections of spatially restricted neuron populations in thalamus can provide a functionally relevant level of connectomic analysis, but these need to be integrated within the same common reference space. Here, we present a pipeline for the segmentation, registration, integration and analysis of multiple tract-tracing experiments. The key difference with other workflows is that the data is transformed to fit the reference template. As a test-case, we investigated the axonal projections and intranuclear arrangement of seven neuronal populations of the ventral posteromedial nucleus of the thalamus (VPM), which we labeled with an anterograde tracer. Their soma positions corresponded, from dorsal to ventral, to cortical representations of the whiskers, nose and mouth. They strongly targeted layer 4, with the majority exclusively targeting one cortical area and the ones in ventrolateral VPM branching to multiple somatosensory areas. We found that our experiments were more topographically precise than similar experiments from the Allen Institute and projections to the primary somatosensory area were in agreement with single-neuron morphological reconstructions from publicly available databases. This pilot study sets the basis for a shared virtual connectivity atlas that could be enriched with additional data for studying the topographical organization of different thalamic nuclei. The pipeline is accessible with only minimal programming skills via a Jupyter Notebook, and offers multiple visualization tools such as cortical flatmaps, subcortical plots and 3D renderings and can be used with custom anatomical delineations.

**Supplementary Information:**

The online version contains supplementary material available at 10.1007/s12021-023-09644-4.

## Introduction

The thalamus is the main gateway for information about the world and the body to the cerebral cortex, and a central hub of the sensorimotor, cognitive and arousal networks in the brain (Clascá, 2022). Modelling thalamic function in a biologically accurate manner requires high resolution and reasonably complete data on the neural connectivity matrix between thalamus and cortex. For example, it is crucial to know the topographical rules between each thalamic nucleus (where projection neuron somata reside in thalamus) and its target regions (where projection axons terminate in the cortex). Branching complexity and convergence/divergence of projection axons vary markedly between nuclei. For example, neurons in some nuclei target a single, focal spot in just one cortical area while those from other nuclei can target several cortical areas in a multi-focal or diffusively-spread fashion (Sherman, 2016; Clascá, 2022). Amongst the focally projecting nuclei, the ventral posterior medial nucleus (VPM) of rodents has attracted a lot of attention because of the one-to-one connections of its cells with selective points of the primary somatosensory area (SSp). Amongst these, the "barrel" domains of SSp layer 4 are of particular relevance. Cortical cells in each barrel process sensory information from receptors at the base of one and the same vibrissa (whisker hair). This organization has made VPM a favourite model for experimental studies on the development, physiology and computation of thalamocortical pathways.

The introduction of new imaging (Amato et al., 2016; Gong et al., 2016; Stelzer et al., 2021) and viral vector labeling technologies (Harris et al., 2012) along with the focusing and standardization of connectomic studies on mice of the same age, sex and genetic background (Oh et al., 2014) have in recent years allowed the analysis of the structural connectivity of the brain, and the consistent and additive comparison of data from different experiments and laboratories. These datasets include population and single-cell axon tracing data. These strategies are not without caveats. For example, bulk-labeling tract-tracing experiments are often the result of large injection volumes that provide abundant but ambiguous information on the wiring of the pathways, with a prime example being the Allen Mouse Brain Connectivity Atlas (AMBCA) (Oh et al., 2014). Recently, some strategies have been developed to restrict the labeling to specific groups of cell populations within the injected area through the use of Cre-dependent vectors (Harris et al., 2019). In recent years there has also been a shift towards reconstructing single cells, prompting the release of large numbers of reconstructed thalamic neurons (Winnubst et al., 2019; Peng et al., 2021). That said, hundreds of additional neuronal reconstructions would be needed to achieve a coverage of thalamic nuclei similar to the AMBCA (see Table 1 for more information).

However, much smaller, selectively localized groups of projection neurons could provide information that is easier to analyze and more functionally relevant as these neurons share to a large extent the same inputs (Casas-Torremocha et al., 2022; Acsády, 2022). To produce small compact clusters of thalamic projection neurons that are crucial to bridge the gap between nucleus- and single cell-level connectomic data, tracer delivery through microiontophoresis is an ideal method. Several substances such as biotynilated dextranamine (BDA) or *Phaseolus vulgaris* leuco-agglutinin (PHA-L), or even electrically-charged viral vectors can be used to this end (Wang et al., 2014; Mukherjee et al., 2020). Over several days after the microinjection procedure, the cells in the injection volume (containing the somata) actively transport the marker substance all over their axonal arbor up to its most distal branches. By adjusting the injection parameters, the injection volume (containing the somata) can be made very small, in the order of tens of cells. Visualization of the labeling with these techniques usually requires serial tissue sectioning and selective staining. These procedures may introduce physical distortions and misalignments of the axon segments contained in the various sections. In addition, the analysis of the labeling and their optical microscopy is usually limited to the examination of 2D sections, making it difficult to compare the micropopulation data between different laboratories, thereby substantially restricting their reusability.

Therefore, registration of neuroanatomical data to a 3D coordinate system can add spatial context to multiple modalities and experimental measurements, thus allowing them to be subjected to spatial statistical analyses, as well as 3D visualization and predictive modeling. These analyses can lead to finding meaningful links between the different modalities and augment anatomical brain models. Recently, the Allen Mouse Common Coordinate Framework (CCF) (Wang et al., 2020) has emerged as the gold standard reference template. Built by averaging serial two-photon (STP) volumes of 1675 adult mouse brains in 10 $\mu$m isotropic resolution across both hemispheres, it constitutes the most complete 3D template of the mouse brain to date.

However, the registration is in itself a complex process that can be approached using two complementary approaches. The first approach is to register the template to the experimental data, which has the advantage of providing anatomical context to the data while preserving their spatial context. As a prime example, the Human Brain Project has developed robust software tools for the registration of the CCF template to mouse brain histological sections. Integrated into the QUINT workflow (Yates et al., 2019), these tools register template section images to experimental section images, segment out useful objects such as cell bodies or neurites and quantify the counts of these objects across the corresponding brain regions, as delineated by the Allen Reference Atlas (ARA).

That said, the registration of template section images to experimental ones makes it impossible to maintain a common reference space across multiple tracing experiments. Therefore, a second approach is to register the experimental data to the template, which preserves the spatial context of the template while altering the spatial context of the experiments. This is necessary for addressing questions which require the integration of multiple experimental measurements or modalities, such as the construction of a somatotopic map or a connectivity matrix. Note that both approaches can be applied either in a direct 3D volume to 3D volume fashion, or in a 2D fashion in the case of experimental section images. In the latter case, this is achieved by representing the reference template as an ensemble of 2D template section images, followed by finding the template section corresponding to each experimental section and then performing the registration for each image pair.

**Fig. 1:**
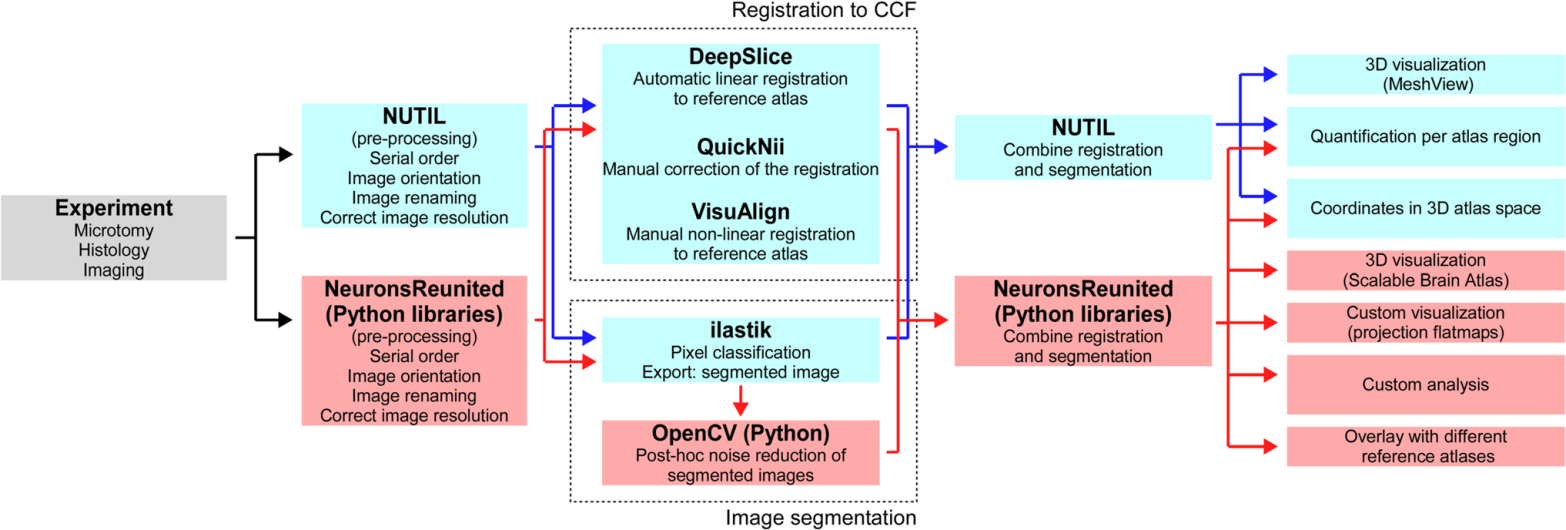
A schematic illustration of the pipeline presented in this work and in parallel its comparison to the QUINT workflow. The top sequence of blue boxes describe the step-by-step procedure of the QUINT workflow from experiment to the final quantification of the analysed results in terms of brain region statistics and 3D atlas coordinates. The bottom sequence of light red boxes describe the step-by-step procedure of our pipeline, which have the same starting point but the intermediate steps have been modified and there are two additions in the final step. The major difference is the replacement of the Nutil software with Python-based libraries for pre- and post-processing of the experimental image sections before and after registration and segmentation. In addition, two new tools have been added to aid registration, one Python-based library has been added to aid segmentation, and a number of visualization tools have been incorporated to aid the visual inspection of the final results. A substantial part of the Methods section is dedicated to describing the motivation and implementation details of these divergent steps

Given our goal of combining multiple experiments, we follow the second approach in this work. We invert the registration step of QUINT by registering experimental section images from tract-tracing experiments to the CCF and then we quantify the injection volume and neurites across CCF. The advantage is that the registered data can now be integrated with other data, such as tract-tracing experiments from the AMBCA and morphological reconstructions of single-neurons, or used with updated brain parcellations (Fig. 1).

## Methods

### Animal Handling and Experimental Procedures

**Fig. 2:**
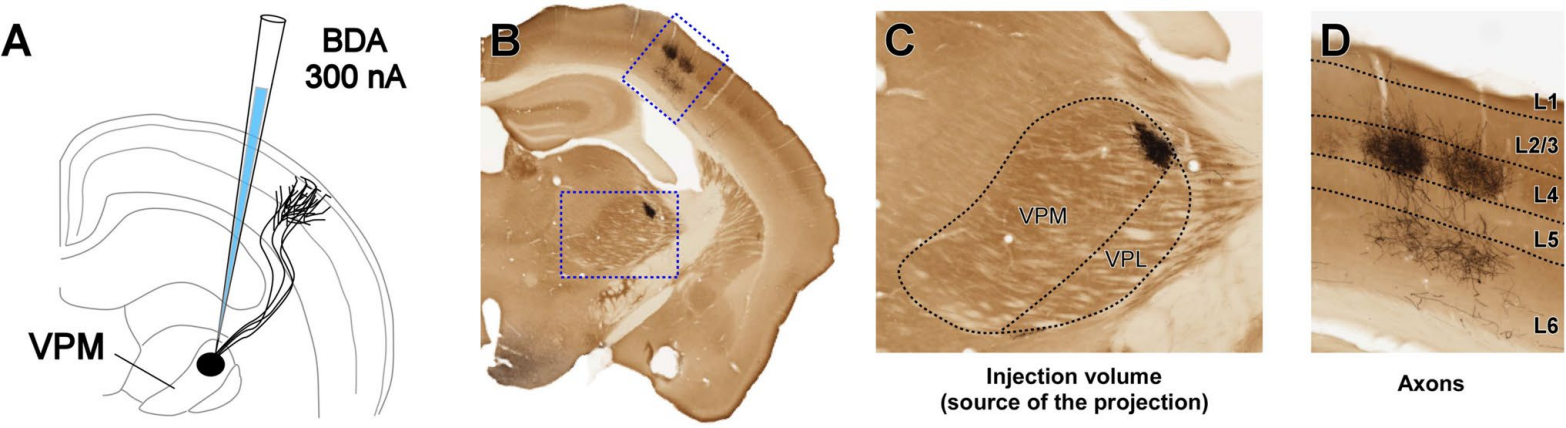
Labeling procedure of small populations of long-range ipsilaterally projecting thalamic neurons in VPM. **A** 10kDa biotinylated dextranamine (BDA) was iontophoretically injected into VPM (0.3 $\mu$A, 1 s ON/OFF, 45 min). After 7 days, the animal was sacrificed, perfused transcardially with the brain removed, postfixed overnight, cryoprotected by soaking in 30% sucrose and sliced coronally on a freezing microtome. **B** Example of an experimental section. The sections were processed using avidin-biotin-peroxidase and diaminobenzidine-glucose oxidase with nickel enhancement, and counterstained with cytochrome oxidase histochemistry. The sections were captured in Neurolucida at 10x magnification. **C**, **D** Higher magnification images of the neuron population labeled in the thalamus (**C**) and their axonal projections to the barrel cortex (**D**)

Micropopulation labeling experiments were performed on adult (60-120 days old, 25-35 g body weight) wild-type C57BL/6 male mice. Animals were bred in the Animal Facilities of the School of Medicine of the Autónoma de Madrid University. All procedures involving animals were conducted under protocols approved by the university Ethics Committee and the competent Regional Government agency (PROEX175/16), in accordance with the European Community Council Directive 2010/63/UE. Animals were housed under standard colony conditions with food and water ad libitum under a 12/12 h light/dark cycle. In total, seven mice were used to obtain micropopulation data (Fig. 2) by means of axon labeling using a 10kDa biotinylated dextranamine (BDA) anterograde tracer.

The anesthetic procedures consisted of intraperitoneal injection of ketamine (0.075 mg/g body weight) together with xylazine (0.02 mg/g body weight), and subsequent maintenance of anesthesia throughout the surgical procedure with isoflurane (0.5 -1%) in oxygen. Ibuprofen (120 mg/l) was added to the drinking water to ensure analgesia during the postoperative period. At the time of sacrifice, animals were overdosed with sodium pentobarbital (0.09 mg/g body weight, i.p.). We targeted the VPM at the centroid defined by the anterior-posterior (AP) $-$1.79, medial-lateral (ML) ±1.75 and dorsal-ventral (DV) $-$3.00 coordinates using a stereotaxic frame (Kopf Instruments) (Paxinos & Franklin, 2019), to which we iontophoretically injected lysine-fixable 10 kDa biotinylated dextran amine (BDA; Invitrogen; 3% w/v solution in 0.01 M PB, pH 7.4) with a 4-15 $\mu$m tip diameter, 0.3 $\mu$A current intensity, 45 min long injection period comprised of 1 s ON/1 s OFF cycles (Fig. 2A). The current was applied using a dual current 260 source (World Precision Instruments, WPI). Animals were then allowed to recover from anesthesia, were returned to their cages and were euthanized after 7 days. Animals were perfused transcardially with 30 ml of saline, followed by 100 ml of 4% paraformaldehyde (PFA; diluted in 0.1 M PB, pH 7.4). Brains were removed from the skull and postfixed overnight at 4 ^∘^C in the same solution. Subsequently, brains were cryoprotected by placing them in 30% sucrose in 0.1 M PB, at 4 ^∘^C, for 48 h.

Brains were freeze-sectioned in the coronal plane at 50 $\mu$m, and sections were collected in two parallel series. Both series were treated as follows: after peroxidase activity blocking by incubation in H_2_O_2_ 0.66% (w/v) in 0.1 M PB for 15 min, sections were incubated for 2 h in avidin-biotin-peroxidase (1:100; Vectastain Elite, Vector Laboratories) diluted in 0.1 M PB. After washing, peroxidase was visualized using the glucose oxidase-3-3’diaminobenzidine (DAB; Sigma-Aldrich) nickel sulfate-enhanced method (Shu et al., 1988). One series was counterstained with cytochrome oxidase (CyO) histochemistry (Wong-Riley et al., 1978), and the other with thionine blue diluted 1:10 for cytoarchitectonic localization of the labeling. Both series were mounted onto gelatin-coated glass slides, air dried, dehydrated in graded ethanol, defatted in xylene and coverslipped with DePex (SERVA Electrophoresis GmbH, Heidelberg, Germany).

### Data Acquisition

Our use case consists of a series of 50 $\mu$m thick evenly-spaced histological sections from the mouse brain, in which a BDA anterograde tracer was injected in the thalamus to study the connectivity between the ventral posteromedial nucleus (VPM) and the somatosensory areas (Fig. 2A). This resulted in the labeling of 10-100 closely located cell bodies and their complete axonal arborizations. For each experiment, we acquired 40 $\mu$m-thick stacks of images with a z-step of 5 $\mu$m covering whole sections (20-25 $\mu$m after shrinkage) at 10x magnification using a Neurolucida platform (MBF Microsystems, Willinston, VT, USA) mounted on brightfield microscope (Nikon Eclipse 80i). Only the series counterstained for CyO were analyzed. Minimum-intensity projection (mIP) images were produced from the stacks, in order to see the location of the labeled somata and axonal projections of the population in the thalamus and cortex, respectively (Fig. 2B-D). The mIP images were cropped so that only one section is shown on the images.

**Table 1:** Hyperlinks for websites, tool descriptions and format descriptions related to our analysis. See main text for details

Repository of our Code on the EBRAINS Collaboratory	https://wiki.ebrains.eu/bin/view/Identity/#/units/all:projects:hbp_pp:neuronsreunited
Repository of our Code on Github	https://github.com/ntimonid/Population_Integrator
NeuronsReunited cortical flatmap viewer	https://neuroinformatics.nl/HBP/allen-flatmap/
Allen Institute for Brain Science	https://alleninstitute.org/
Allen Software Development Kit	https://allensdk.readthedocs.io/en/latest/
AMBCA repository (Oh et al., 2014; Harris et al., 2019)	https://help.brain-map.org/display/mouseconnectivity/Documentation
CCF v3.0 (Wang et al., 2020)	http://connectivity.brain-map.org/3d-viewer
Enhanced and Unified Anatomical labeling (Chon et al., 2019)	https://kimlab.io/brain-map/atlas/
Mouselight database (Winnubst et al., 2019)	https://ml-neuronbrowser.janelia.org/
Braintell database (Peng et al., 2021)	https://braintell.org/seu-allen/index.html
Cortical flatmap templates (Knox et al., 2018)	https://download.alleninstitute.org/informatics-archive/current-release/mouse_ccf/cortical_coordinates/ccf_2017/
Jupyter Notebook	https://jupyter.org/
NumPy	https://numpy.org/
Matplotlib	https://matplotlib.org/
NIfTI files	https://nifti.nimh.nih.gov/
JSON files	https://en.wikipedia.org/wiki/JSON
SBA Composer (Bakker et al., 2015)	https://sba-dev.incf.org/composer/index.php
VisuAlign software	https://www.nitrc.org/projects/visualign
QuickNII software (Puchades et al., 2019)	https://quicknii.readthedocs.io/en/latest/
Ilastik software (Berg et al., 2019)	https://www.ilastik.org/
OpenCV library (Bradski, 2000)	https://opencv.org/
Neurolucida software	https://www.mbfbioscience.com/products/neurolucida
Extensible 3D script library	https://www.x3dom.org/
DeepSlice web-based tool (Carey et al., 2022)	https://www.deepslice.com.au/

### Step-by-Step Description of the Pipeline

The pipeline consists of a Jupyter Notebook, which is an open source web application (see Table 1 for a hyperlink to detailed description), together with a number of supporting script modules written in the Python programming language. The Notebook is structured as a mixture of documents, termed cells, which can be used to execute live code or be readable in text format that contain the respective code descriptions or results. In the case of this work, consecutive live-code and readable cells correspond to executable steps of the pipeline and their descriptions, respectively, in the serial order that is described in the succeeding paragraphs (Fig. 1). For a minor fraction of steps that are not executed inside the pipeline, we will further elaborate on their respective subsections.

**Fig. 3:**
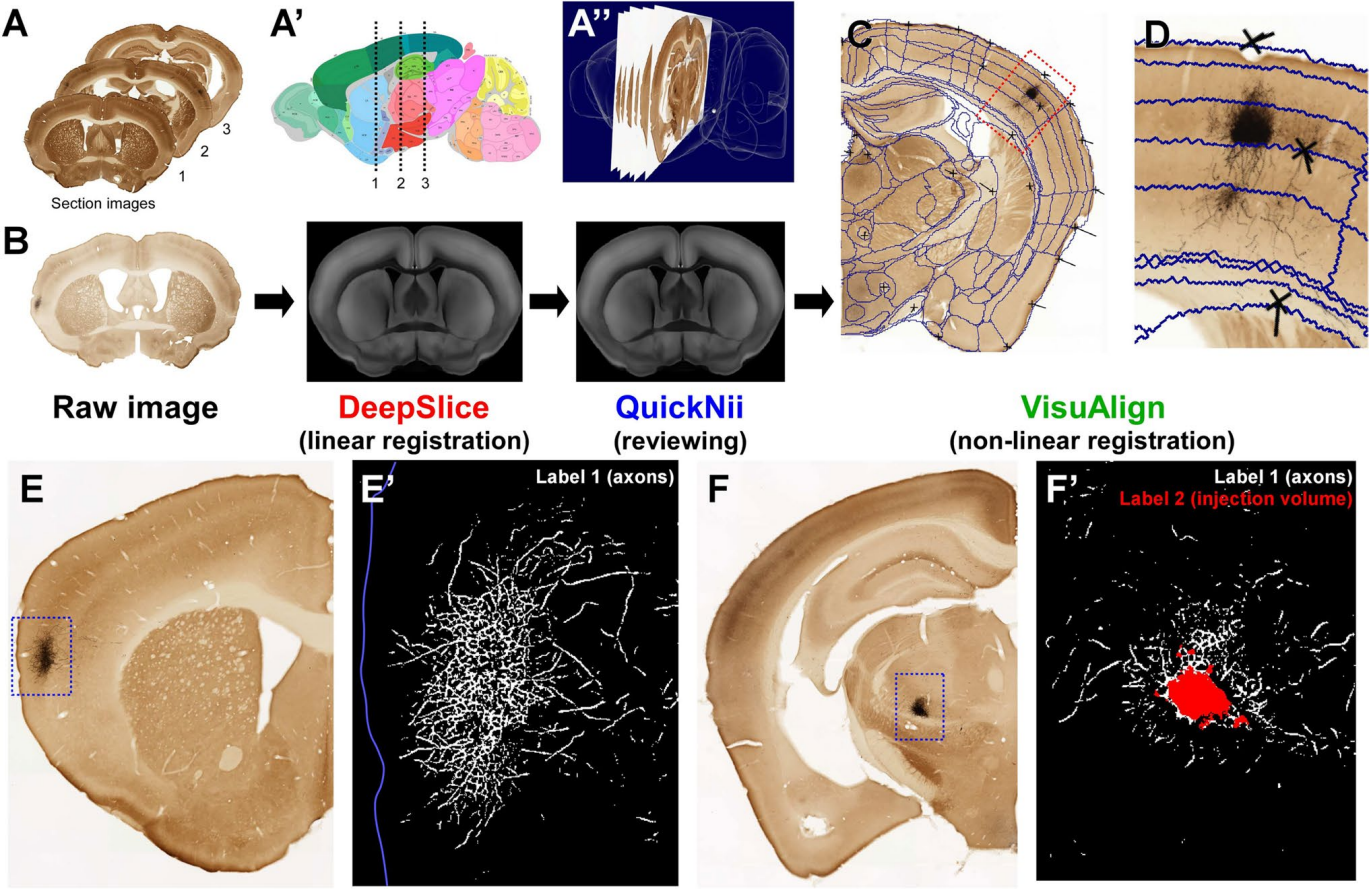
Registration of the images to the Allen Common Coordinate Framework (CCF) and segmentation with ilastik. **A**-**A”** A collection of coronal histological sections (**A**) has to be registered to their correct anatomical position in the brain (**A’**), as defined by the Allen CCF template (**A’’**). In order to achieve this, both linear and nonlinear corrections are applied to the images. **B** For each of the input images (**B**, left), the DeepSlice algorithm automatically finds the most similar section from CCF (**B**, middle). The result is manually assessed and corrected using the QuickNII software (**B**, right). **C** For the manual non-linear registration, the VisuAlign software is used to place markers on regions, as corresponding points between the histological section and the most similar CCF section, which it then drags to deform the latter until it fits the shape of the former. **D** Higher magnification image showing the laminar distribution of the arborization. The corresponding points (markers) are represented by crosses. Note the precision of the correspondence between layers and their delineations in the atlas. **E**-**E’** The section images (**E**) are segmented using ilastik’s Pixel Classification workflow to delineate the labeled neurites (label 1, white) and their respective somata (label 2, red) from the background (**E’**). **F**, **F’** Example of a section containing the labeled population in the thalamus (**F**). The first label (axons) corresponds to segmented axons and the second label (injection volume) corresponds to segmented somata (**F’**)

The following steps are applied iteratively to each batch of mIP images belonging to a particular tracing experiment, until all the sections of the experiments under analysis have been segmented and registered to CCF (Fig. 3A’-A”).

#### Data Pre-processing

Prior to any pre-processing, we first assign to each image a unique three-digit number that sorts them along the anterior-to-posterior axis of the brain, which is a prerequisite for the successful processing by the registration tools. For instance, the first section image will receive the number s001 to the end of its filename, where the symbol ’s’ stands for section.

The first pre-processing step is to downsample the experimental section images to satisfy the requirement of QuickNII for input images smaller than 16 megapixels. The downsampling factor is the same for all the sections that belong to a specific experiment. So if there are images from rostral parts of the brain that can keep 1 out of every 4 pixels and images from caudal parts that can only keep 1 out of every 5 pixels, then the downsampling is performed at 1 out of 5, so that they are all at the same resolution. We downsample each image by selecting every $n^{th}$ pixel in each direction, where n is the highest value calculated by: $\sqrt{\frac{height \mathop{\times} width}{15 \mathop{\times} 10^6}}$.

The second step is to rotate and/or mirror images that have been characterized by the user as requiring these operations. For instance, some sections could be mounted onto the slide in the wrong position, or could be flipped horizontally.

#### Registration to CCF

First, linear registration is performed automatically using DeepSlice with the aid of QuickNII. DeepSlice is a deep neural network trained to automatically register coronal section images to CCF v3.0 (Carey et al., 2022) and QuickNII is a software tool for performing semi-automated affine registration of section images to CCF (Puchades et al., 2019) (see Sections S1.2.1, S1.2.4 for more details on both tools). Thus, as a first step, the experimental section images are registered automatically by DeepSlice, which returns an xml file storing the required affine transformations as a set of anchor vectors. This file is then loaded in QuickNII, and the performance of DeepSlice is assessed and corrected manually by neuroanatomical experts (Fig. 3B). This is done for each experiment following the guidelines provided by the QuickNII documentation (see Table 1).

Non-linear registration is then performed completely manually using the software tool VisuAlign (see Table 1), in order to refine the linear registration obtained by DeepSlice and QuickNII. VisuAlign provides a graphical user interface that, for every experimental section, overlays its corresponding template section. This allows the user to stretch the overlay and place histological landmarks in order to find corresponding points between the two. This results in the template section being non-linearly transformed to fit the experimental section (see Section S1.2.3). The output of VisuAlign is a json file similar to the xml file obtained from QuickNII, that stores the original anchoring vectors and the sets of corresponding points between the experimental section and the template section (Fig. 3C, D).

#### Image Segmentation

The pipeline then proceeds with segmenting out the objects of interest (somata and neurites) of the labeled population from the image, by executing a classifier trained using ilastik’s pixel classification workflow (Berg et al., 2019) (Fig. 3E-F). For a technical description of how an ilastik-based classifier is built, see Section S1.2.2. The training was performed using a number of downsampled whole-section images that were manually selected from the registered datasets to cover the whole range of labeling and staining conditions of the sections. All features offered during the "feature selection" stage of ilastik’s pixel classification workflow were selected (Gaussian smoothing, Laplacian of Gaussian, Gaussian Gradient Magnitude, Difference of Gaussians, Structure Tensor Eigenvalues, Hessian of Gaussian Eigenvalues) for all possible neighborhood sizes in pixels: (0.3, 0.7, 1.00, 1.60, 3.50, 5.00, 10.00).

The classifier was trained to segment the image pixels into three categories: (1) neurites or axon projections stemming from the labeled population, which appeared as thin processes when isolated, and as dark blobs when converging on a single spot; (2) injection volumes that contained the somata that were the source of the axonal projections, which presented themselves as extremely dark, relatively large areas; and (3) background. Since the visual features of these three categories were usually spatially non-overlapping and very different from each other feature-wise, no further analysis (such as object classification) was required. The only exception was when a large number of axons converged on a very specific region, resulting in dense arborizations that looked similar to injection volumes. However, this issue is automatically resolved in the post-processing steps of the pipeline, which is described in the paragraphs below. Lastly, the classifier was also trained to avoid misclassification issues that resulted in false positives when dealing with different types of histological artifacts (such as changes in axonal signal or background intensity, bubbles, tears in the glass, red blood cells due to incomplete perfusion, fibers caught under the coverslip, etc.); this was achieved using synthetic png images containing cropped snippets of a section in which the artifact was present. Cropping was performed to reduce memory use. The output of this step was an .ilp file containing both the trained classifier and the images. This file can be used to segment any experiment performed under the same conditions as those of the training dataset.

Ilastik can be used automatically in headless mode, which utilizes a pre-trained classifier without the need of a graphical user interface for segmenting the input images. The communication of Python with ilastik is mediated through the execution of shell script (UNIX-based operating system) commands, which instruct ilastik which section images to use as input to the segmentation classifier.

Following the completion of an image segmentation, the classifier will store the segmented image in the same directory as from which the image was parsed, with the output image having the same filename as the original one but additionally including the extension "SimpleSegmentation". Since ilastik stores the label value for a given pixel as an integer ranging from 1 to n, where n corresponds to the number of labels, we have added an additional post-processing step for improving the visual distinction between the different labels in the image. Given that the resulting image is gray-scale, we change the values corresponding to the unique label ids to have a greater distance between them in gray-scale. Therefore, we change the value 1 of the axonal segments label to 255 (white), the value 2 of the background label to 0 (black), and the value 3 of the soma label to 129 (gray).

We then clean the segmented images by removing potentially false positives corresponding to background pixels that have been falsely labeled as somata or axons. This operation is performed by first identifying objects in the image, corresponding to aggregations of pixels that are labeled as soma or axons. The number of maximum orthogonal steps needed to consider a neighboring pixel as part of the same object is 3 and it is estimated by taking the squared euclidean distance between the two pixels. The operation is then followed by classifying the objects with less than 12 pixels as false positives. Pixels satisfying these criteria will be assigned the value 0, hence relabeled as part of the background.

#### Reverse Registration of the Segmented Images

**Fig. 4:**
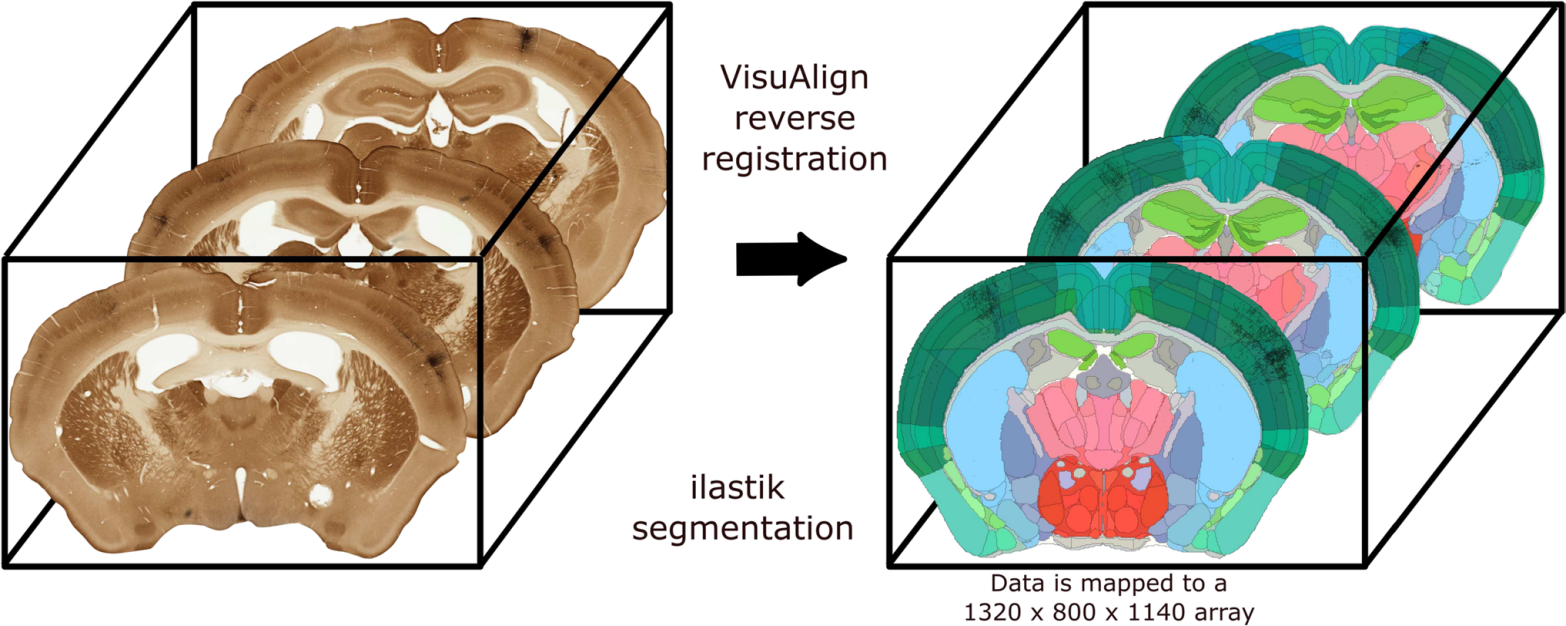
Mapping to the segmented and registered sections to the CCF brain template to create a 3D volumetric representation of the data at a resolution of 10 $\mu$m. Left: a sequence of raw experimental sections which have been aligned from bottom to top along the anterior-posterior axis. Right: the corresponding sequence of registered and segmented populations from the left panel, which have been spatially overlaid on the ARA parcellation of the mouse brain and are likewise aligned along the anterior-posterior axis. The colours on the right panel represent anatomically distinct brain areas of the brain, as defined by ARA, whose boundaries are shown by black lines. The registration of populations to the CCF is achieved through the reverse non-linear registration obtained from the VisuAlign software

Subsequently, we apply the reverse non-linear registration of each segmented image to CCF (see Section S1.2.3 for the technical description), in order to map the labelled pixels to the 3D brain template (Fig. 4). The trick here is that we register the cleaned segmented images directly instead of the raw experimental ones, which is allowed since they are the same size. This trick allows to promptly and directly embed the axonal and somatic segments of the analysed experiment into the 3D coordinate space of CCF, without including any additional and unnecessary histological information. The reverse registration is computed using the back-end Python libraries of VisuAlign.

We use the transformation formula that is outputted by QuickNII (Puchades et al., 2019) (see Section S1.2.1, Eq. S1) for converting pixel coordinates to the 3D voxel coordinates of the corresponding points in CCF. Given that the highest possible CCF resolution is 10 $\mu$m, which can be lower than the section resolution, we count the number of segmented pixels that have been registered to a given atlas voxel and we quantify the voxel using this number. For instance, a voxel with the value 100 reflects 100 segmented pixels that fall within the corresponding cubical volume. As an intermediate curation step, voxels outside the VPM containing pixels that have been labeled as part of the injection volume, are considered to be false negatives of neurite pixels and are thus relabeled as neurites. This is possible due to the a-priori knowledge of the correct anatomical location of the injection volume.

We create dictionaries storing the voxel coordinates of the labeled axons and somata, respectively. The coordinates have the same voxel resolution, orientation and origin as the 10 $\mu$m Allen Reference Atlas (ARA). Hence, the outcome of registering the entire image stack into CCF is a source and a target dictionary containing the coordinates of the somatic and axonal segments separately. The volumetric space of 10 $\mu$m ARA, in which the coordinates of the two dictionaries reside, has the dimension of 1320 voxels in the x-axis, corresponding to the Anterior-Posterior axis, 800 voxels in the y-axis, corresponding to the Superior-Inferior axis, and 1140 voxels in the z-axis, corresponding to the Left-Right axis, while the origin of orientation corresponds to the Anterior-Superior-Left corner of CCF.

Thalamocortical projections to the cortex are exclusively ipsilateral. For that reason, we take advantage of the symmetry of CCF (Wang et al., 2020) to focus only on the left hemisphere in order to reduce the memory required for the visualizations. Therefore, we split the source and target dictionaries into two new dictionaries each corresponding to one of the two hemispheres. To be able to visualize everything on the same hemisphere, the coordinates belonging to the original right hemisphere are now inverted along the Left-Right axis and are thus mapped to the left hemisphere.

#### Overlay with Different Reference Atlases

In order to be able to evaluate the anatomical accuracy of the entire registration procedure, we superimpose each registered segmented section onto the corresponding template section. We first use the spatial coordinates of the template section to reconstruct it as an image, in which each anatomical area has been assigned a certain colour. See (Puchades et al., 2019; Yates et al., 2019) for how it was first constructed as part of QUINT. We then delineate the boundaries of each anatomical area using black lines, in order to improve the visual distinction between areas. Finally, we overlay the segmented populations onto the delineated template section using the colour black. Each overlaid section image can be stored as an scalable vector graphics (svg) file to be exported for the user.

**Fig. 5:**
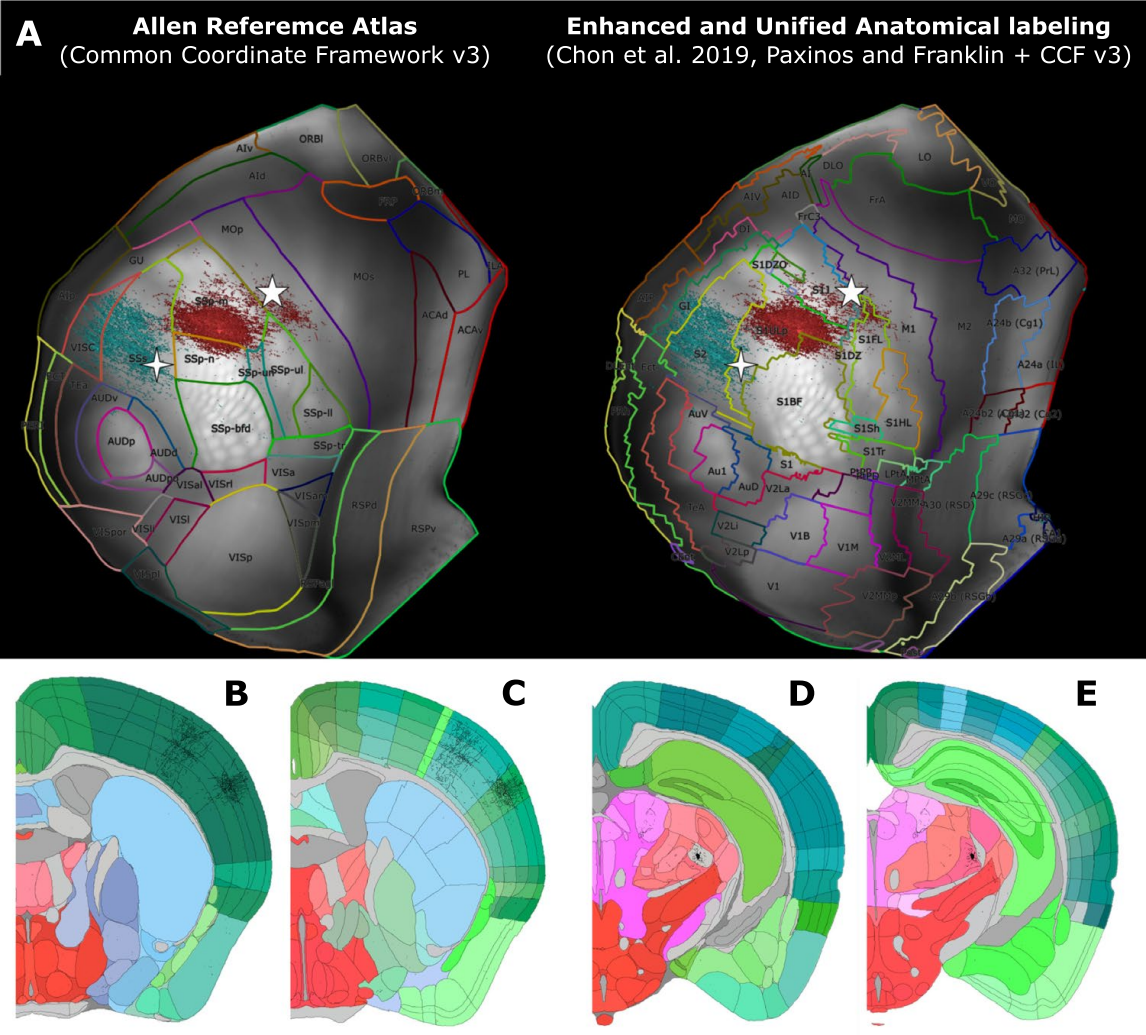
The coronal section and cortical flatmap visualizations can be performed using the anatomical parcellation of different reference atlases in order to provide multiple possible anatomical interpretations of the results. **A**, **Left** Cortical flatmap showing the Allen Reference Atlas (ARA) parcellation. **A**, **Right** Cortical flatmap showing the Enhanced and United Anatomical labeling (EUAL) parcellation, which integrates the ARA and the Paxinos & Franklin atlas according to the Common Coordinate Framework v3.0. In addition to the differences in nomenclature and general shape of the cortical subdivisions, note the discrepancies in the border of MOp/M1 and SSp/S1 (white asterisk, *) or SSp/S1 and SSs/S2 (white + sign). **B**-**E** Coronal sections from two different experiments delineated with the ARA (**B**, **D**) and EUAL (**C**, **E**) parcellations. The first coronal section (**B**, **C**) exhibits axonal segments and the second section (**D**, **E**) exhibits soma injection volume. The interpretation of the anatomical distribution of the labeled axonal segments or somata slightly changes by the parcellation that is used. **B**-**C** A group of axonal projections targeting the primary somatosensory nose (SSp-n) and barrel field (SSp-bfd) in ARA, is located on the anatomical border between the two areas, whereas they correspond to exclusive projections to the barrel field (S1BF) in EUAL. Another group of axonal projections targeting the supplemental area (SSs) in ARA correspond to projections in the upper limb (S1UL) in EUAL. **D**-**E** A population of labeled somata is located either in the auditory radiation of the thalamus or in VPM depending on the ARA or EUAL parcellation, respectively

As an additional anatomical parcellation alongside ARA, we utilize the Enhanced and United Anatomical labeling (EUAL) delineation in two ways. First, the above described procedure is repeated for template sections corresponding to both the ARA and the EUAL delineations (see Table 1 for the respective websites), which often show discrepancies (see Fig. 5B-E for two examples). Our motivation for this option was to provide different versions of anatomical delineation to the user in order to allow them to draw their conclusions when observing the various differences in anatomical delineation and nomenclature without emphasizing only one possible interpretation of the mouse reference space (see Section S1.4) (Chon et al., 2019).

Second, we create a similar overlay for dorsal and top-view cortical flatmaps (see Fig. 5A for an example and "Custom Visualizations" for further explanation). Both types of visualizations are used to compare the anatomical boundary delineations from the ARA and EUAL labels, both on the local scale of assessing a coronal slice under registration and on the global scale of assessing the cortical surface of the whole volume following registration.

### Extension to Multiple Populations

The steps described in the above paragraphs of this section can be applied to multiple experiments registered with a single-run of the pipeline. If more than one population is registered using the pipeline, then the source and target dictionaries are extended to include multiple populations which will be distinguished by their experiment ids. To be able to visualize multiple registered populations together, we have to ensure that the populations can be visually distinguished from one another and that there will be a certain rule dictating the appearance of the data points when there is anatomical overlap between two or more populations at the same spatial coordinate. To deal with these two issues, we implemented a colour-coding strategy; we first assign a unique colour from the RGB scale to each population so that it can be identified in any form of visualization within the pipeline. When there are multiple populations at a given spatial coordinate, it will be coloured according to the population having the highest number of segmented pixels within it (Fig. 7H). This colour-coding strategy aims to emphasize the spatially-specific source location area of the somata and the target location areas of their dominant projections.

### Custom Visualizations

**Fig. 6:**
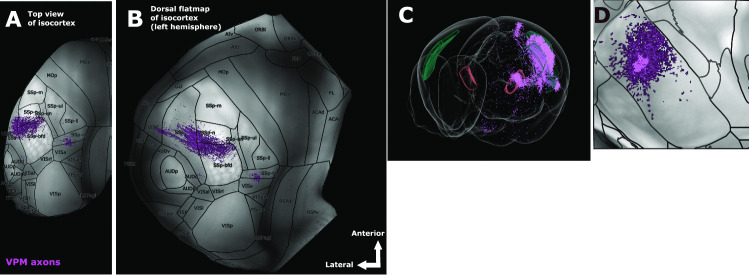
Custom plots for data visualization, using a registered population experiment as an example. **A** Top view of the isocortex showing the distribution of thalamic axons that are originating from VPM. For a given point in space, the intensity of the colour reflects the number of detected axonal segments, as measured by the number of pixels in the corresponding registered and segmented section image that have been labeled as axons. **B** A dorsal flatmap of the left hemisphere of the cortex, illustrating the same projection as in **A**. The arrows in the bottom right corner of the figure indicate the spatial orientation of the flatmap along the anterior-posterior and lateral-medial axes. **C** Integration with the Scalable Brain Atlas Composer tool (Bakker et al., 2015) enables a number of 3D visualization options. A 3D rendered model of the mouse brain template based on CCF is used to embed the registered population in the form of a 3D point-cloud. **D** A visualization of the VPM is constructed by estimating three overlaid layers of maximum projection plots onto the coronal plane intersecting the VPM area. First layer: anatomical border delineation, which is drawn by black contour lines generated by the boundaries between different subcortical nuclei, as defined by the ARA parcellation. Second layer: intensity of the gray matter, as defined by the STP volumes provided by (Wang et al., 2020). Third layer: somato-dendritic distribution of the population in purple

Following the complete registration of one or more experimental datasets, a number of visualization strategies are implemented. To visually inspect the topographical organization of the registered populations, we produce a number of 2D cortical flatmaps and subcortical visualizations. We first create and plot a dorsal cortical flatmap (Knox et al., 2018) that overlays the various registered populations using the aforementioned colour-coding strategy (see Fig. 6A-B for an example and Section S1.3 for the technical description). Four flatmaps are plotted, the first two correspond to the two hemispheres superimposed on the left hemisphere. The latter two repeat the same procedure but with the anatomical boundary delineation of the EUAL atlas instead of the ARA. The motivation for showing both types of anatomical delineation overlays is the same as the one given for individual section images (Fig. 5A).

It is currently not feasible to represent a subcortical nucleus such as VPM in a flatmap due to the lack of a proper characterization of the barreloid structures. If a proper parcellation of the barreloids were to be established, we could in principle develop a VPM flatmap that would be defined by the dorsomedial-to-ventrolateral direction of the barreloids. As a temporary substitute until a proper parcellation, we instead create three maximum projection plots corresponding to the coronal, sagittal and horizontal plane respectively, as a surrogate subcortical visualization of the soma distributions of the registered populations. Each subcortical visualization comprises an overlay between three maximum projection plots along the given axis: the soma locations of all populations, which are colour-coded in the same fashion as their axonal patterns, the corresponding gray-matter volume of the Allen average template (Wang et al., 2020), which serves as the background, and the anatomical border delineation of VPM that has been drawn based on the ARA parcellation using contour lines generated from the borders between the different nuclei (Fig. 6D).

**Fig. 7:**
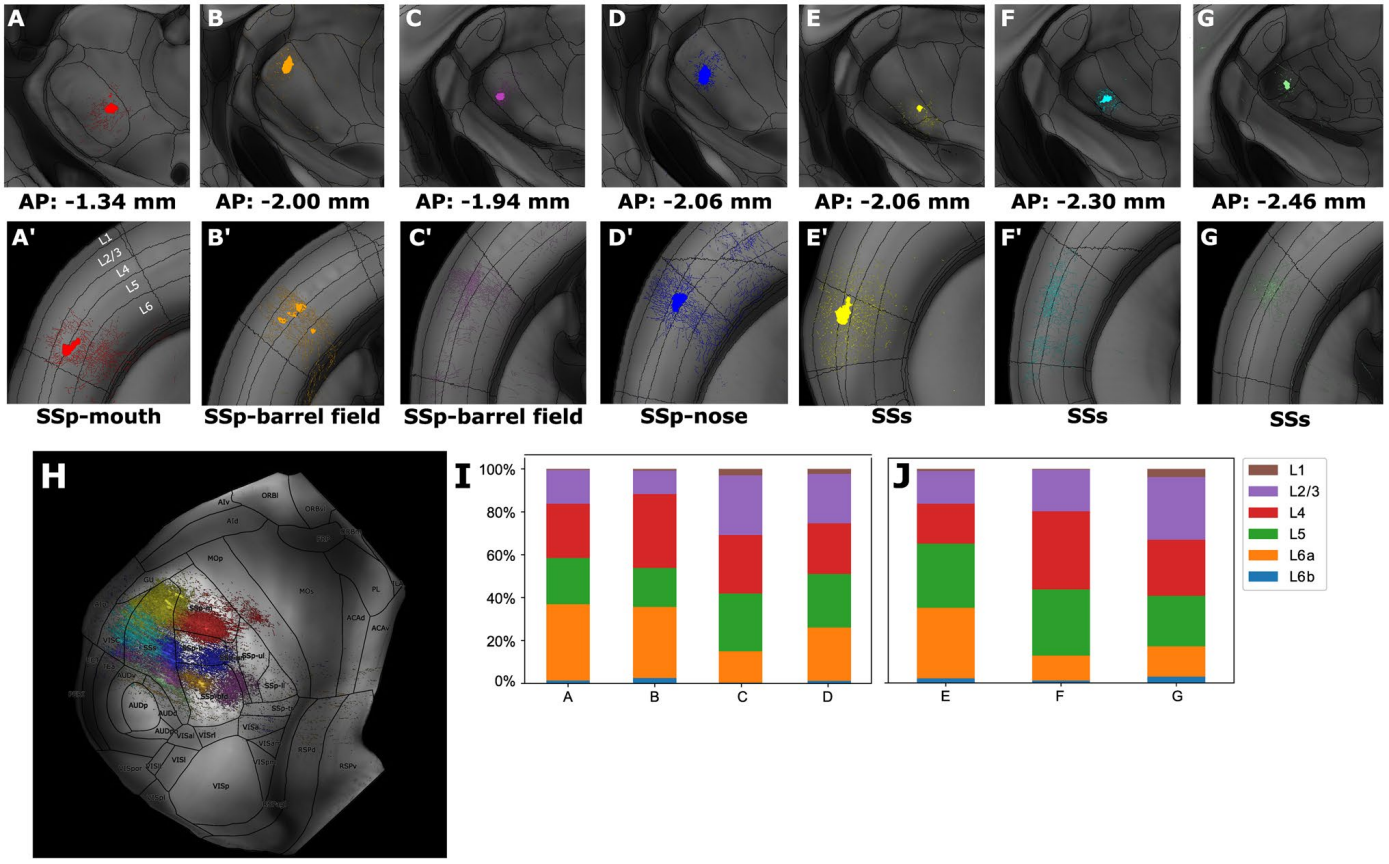
The cortical distribution of VPM axons depends on the position of their soma. **A**-**G** Injected soma locations of seven distinct neuronal populations projecting to the primary (SSp; **A’**-**D’**) and supplemental somatosensory (SSs) area (**E’**-**G’**). The populations correspond to BDA-tracing experiments 1-7 that have been registered by our pipeline. The color-coding and anatomical acronym at the bottom center of each plot are used to distinguish and classify each population by the somatosensory sub-area that it most prominently targets. The raw experimental section images have been registered to coronal template sections of the CCF, organized along the posterior-anterior axis, with their coordinates shown at the bottom of each upper panel. **H** Dorsal cortical flatmap illustrating the topographical distribution of the projection patterns of each population. The color-coding is the same as in **A**-**G**. Details about the flatmap can be found in Fig. 6. **I**, **J** Relative distribution of the number of pixels labeled as axonal segments within SSp and SSs for the different populations. x-label: letter denoting the population in the same order that it appears in **A**-**G**. y-label: percentage of axonal segments in a given layer, which is normalized to the segments of a given population across all layers. For each somatosensory area, we plot only the populations that most prominently target the area. The color-coding is explained at the top right inset and refers to the layers instead of the experiments. A technical description regarding the cortical flatmap structure can be found in S1.3

Additionally, we visualize the axonal distribution of the populations per cortical layer over the target areas of interest, which in our use-case are the primary (SSp) and supplemental (SSs) somatosensory areas. For each target area of interest, we select the populations that most dominantly project into it and we plot their axonal distribution across layers 1-6 (see Figs. 7I-J for examples). As an extra aid to the interpretation of the results, we import the source and target volumes directly into the Scalable Brain Atlas (SBA) Composer 3D visualization tool (see Section S1.5), which allows for flexible and user-friendly renderings of the data in a direct 3D view (Fig. 6C) (Bakker et al., 2015).

### Integrating Additional Modalities

To corroborate the observations drawn from analysing the registered populations, we can spatially integrate additional data modalities and investigate the overlap in their projections: similarly located single-neurons or populations of neurons are expected to have more similar projection patterns compared to distally located ones. Besides tract-tracing experiments of neuronal populations, morphological reconstructions of long-range projecting neurons (LRPN) is a prime modality for testing the integrative capabilities of the pipeline. Since we are interested in comparing both modalities at the level of axons, we first retrieve all LRPN neurons of interest and obtain their axonal terminal branches as shown in Section S1.6.

We then employ a greedy approach to find LRPN morphologies that could be of the cell-type that belongs to the same BDA-labeled population. For a given population, we first use the k-medoids algorithm (Kaufman & Rousseeuw, 1990) to identify the medoid of a population. The population medoid can be considered as the most proximal soma location to the centroid. This allows for biological plausibility, since a medoid is a *de facto* labeled voxel of the injection volume of the population, while a centroid could be a voxel that is labeled as background. With the medoid identified as the most central soma location of the population, we proceed in making an assumption that defines the anatomical volume that is occupied by the population. Since, we do not expect a perfect coverage of a tracing-experiment in labeling the entire population, we assume that there exists a radius from the medoid, within which any point in anatomical space could contain a potential soma-member of the population. We define the radius as the maximum Euclidean distance found between the medoid and another soma-member of the population multiplied by a factor of 1.5 to account for incomplete sampling. Therefore, a LRPN whose soma is within this radius is classified as a potential member of the population and its terminal branches are assigned to be part of the population’s axons.

We repeat this strategy for integrating our population data with the tract-tracing experiments from other databases. The most proximal experiments are selected based on their injection volume locations. The purpose of this comparison is to compare the results obtained from our registered populations with those obtained by the same modality but performed using a different tracer. A test-case of interest is assessing the topographical specificity of BDA tracers compared to AAV tracers, since the latter have been reported to label larger injection sites that can involve more than one nucleus (see "Introduction").

## Results

Using the steps described in the previous section, we registered several neuronal populations labeled in seven different BDA-labeling experiments (experiments 1 to 7). Their CyO-counterstained sections showed a much higher contrast between background and BDA labeling than expected, while still allowing us to differentiate layers and nuclei. This was possible due to the high levels of metabolic and CyO activity of the neurons in both VPM (Haidarliu & Ahissar, 2001) and the SSp barrels and layer 4 of the cortex (Land & Simons, 1985), which made them appear darker in CyO-stained tissue sections.

The registration accuracy of the experiments was double-checked against the thionin-stained tissue series by an expert under the microscope. Despite DeepSlice finding good correspondences between the experimental and the CCF template section images, we observed that the sectioning angles of the horizontal and sagittal planes had to be searched for manually using QuickNII to further improve the registration. We also observed that DeepSlice classification performance was optimal when dealing with sections with a considerable amount of features, such as those featuring the thalamus and its nuclei (e.g. AP $-$1.58), as opposed to more rostral sections in which the striatum was the most prominent structure (e.g. AP 0.50).

In the following paragraphs we will describe results related to the analysis of the seven registered populations.

### VPM Neurons Exhibit Distinct Topographical Organization, Projection Motifs and Laminar Distribution

After registration to the 3D space, we identified a topographical organization of VPM thalamocortical neurons that mirrors that of the cortex. This means that the populations that specifically targeted SSp were located more rostrally in the nucleus (experiments 1-4, Fig. 7A-D), whereas those that targeted SSs were located more caudally (experiments 5-7, Fig. 7E-G). Each of these populations targeted anatomically distinct cortical sub-areas. The populations could thus be further subdivided according to their cortical targets: from dorsal to ventral VPM, the targets in SSp were related to the whiskers, nose and mouth representations, respectively.

VPM targeted not only the primary somatosensory area (SSp), but also the supplemental somatosensory area (SSs) with both specific (targeting one area) and branching motifs (targeting two or more areas) (Fig. 7H), with the specific projection class being more dominant. Branching architectures were found in the region that has been termed ventrolateral VPM (VPMvl), which acts as the thalamic relay for the extralemniscal pathway (Pierret et al., 2000; Zhang et al., 2021).

The estimation of axonal layer distribution across different experiments was consistent with the notion that all cells in VPM belong to the same high-level projection neuron class (Clascá, 2022). Regarding single-target VPM populations to SSp (experiments 1-2), the main contribution was to layer 4 (25% and 34% of the labeled axon voxels, respectively). Smaller arborizations were frequently seen within the same column, but at the border between layers 5 and 6. These, however, were over-represented due to the presence of retrogradely labeled corticothalamic somata (Wang et al., 2014), whose somatodendritic morphology was visible at the infragranular layers (58% and 54% of labeled thalamocortical axonal segments arborized in layer 5 or 6 in experiments 1 and 2, respectively). Regarding branching populations to SSp (experiments 3-4) or SSs (experiments 5-7), we observed similar patterns regarding layers 4 and 5/6a, but with the addition of stronger arborizations in layer 2/3 (27%, 23%, 19%, 27% for the populations from 3,4,6,7 in contrast to 15% for the populations from 1,2,5, see Fig. 7I-J).

This projection to supragranular layers is something that can be seen in other recent descriptions of the pathway (Zhang et al., 2021), and is is likely due to the different functional roles that each of these subregions of VPM play in the somatosensory system. Whereas VPM targets the layer 4 barrels, VPMvl has been described as targeting the septa in-between them. However, the current version of the CCF does not include any of these delineations, so we can not assess this feature here. Moreover, while VPM has been shown to predominantly target SSp, with some populations branching to SSs (Spreafico et al., 1987), the exact location of the SSs projecting domain has not been properly mapped.

### Single Neuron Projections Match Population Tracing Data for the SSp-Targeting Experiments

Topography is an essential feature of how neural connections are established. Because all mice share the same mechanisms of map formation (Dufour et al., 2003), single neurons and tract-tracing population experiments whose somata and injection volume are in close proximity to each other in the common reference space should have similar projection targets. To validate our results, we anatomically overlaid the registered populations reconstructed axonal morphologies of long-range projecting neurons (LRPN) from the Mouselight and Braintell public repositories (Winnubst et al., 2019; Peng et al., 2021).

**Fig. 8:**
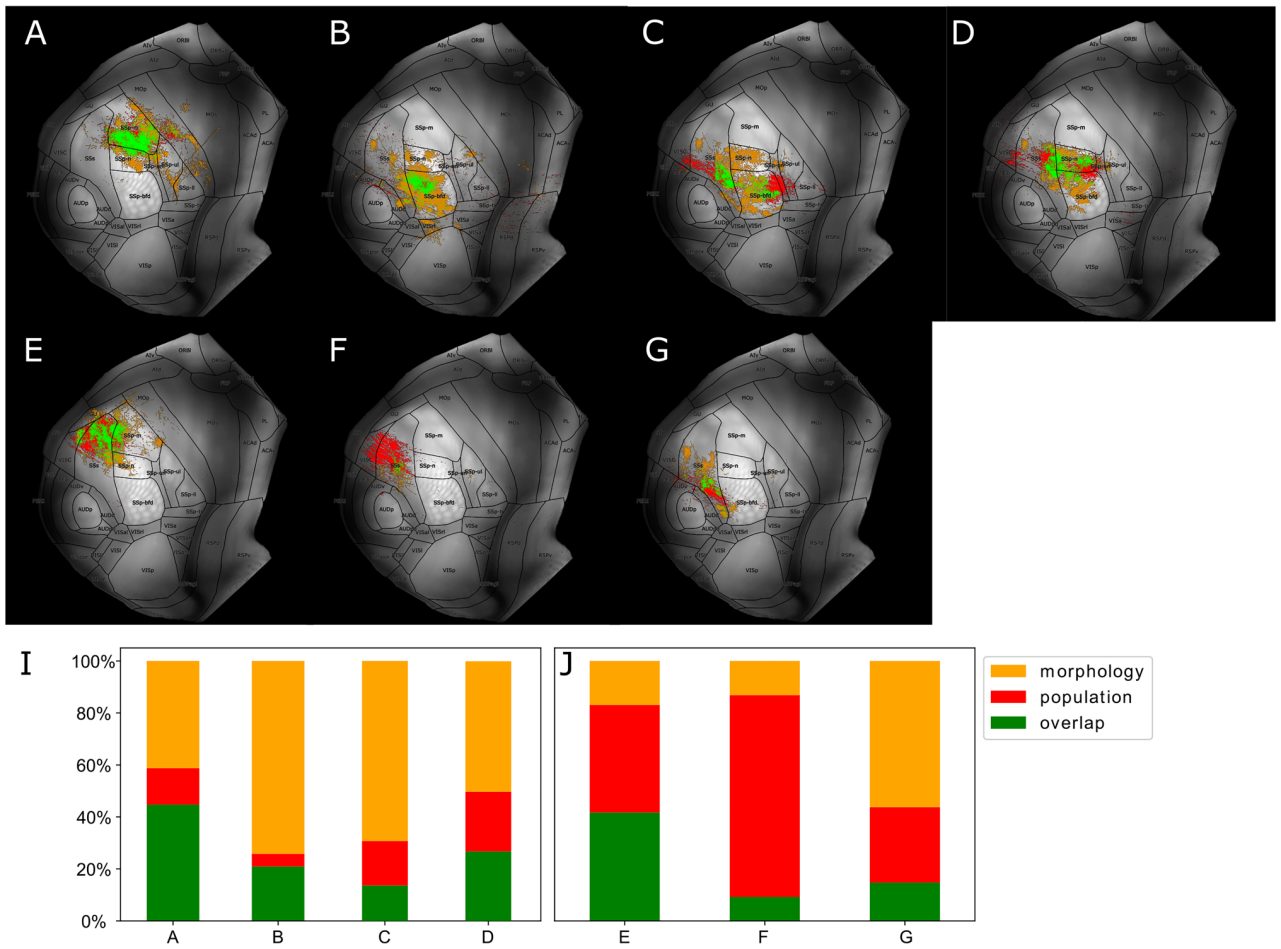
Spatial overlay of registered population experiments with morphological reconstructions of long-range projecting single-neurons (LRPN) in the CCF points to both convergence and divergence between the two data modalities. **A**-**E** Dorsal cortical flatmap representation of the axonal segments of a population, one per plot, overlaid with the axonal terminal branches of those LRPNs, whose soma position is proximal to the medoid of the population somata (see "Integrating Additional Modalities"). The letters **A** to **G** are used to label the populations that correspond to the experiment ids 1-7 and are consistent with the labels used in Fig. 7. A color-coding strategy is used to quantify the three possible spatial overlapping states between the two modalities on the 2D flatmap space: green represents overlap between population and LRPN, red represents exclusive targets by the populations and orange represents exclusive targets by LRPN neurons. **I**-**J** Fraction of points in flatmap space targeted by a population, LRPN or both within SSp (**I**) and SSs (**J**), for the different populations. x-label: letter denoting the population in the same order that it appears in **A**-**G**. y-label: percentage of targeted points that belong to each of the three states, which is normalized by all points within SSp (**I**) or SSs (**J**). For each somatosensory area, we plot only the populations that most dominantly target the area: experiment groups 1-4 and 5-7 dominantly target SSp and SSs, respectively. The color-coding is explained at the top right inset and is consistent with the previous plots. A technical description regarding the cortical flatmap structure can be found in Section S1.3

The anatomical overlay of all BDA micropopulations with proximal LRPN neurons was performed at the level of cortical flatmaps and the VPM maximum projection plots (see "Custom Visualizations" and Section S1.3 for more details). The reason why we compared the modalities in flatmap space and not in the original 3D space was to mitigate potential small registration errors of the data across the cortical column at the scale of micrometers, by averaging the data along the cortical layers. To visualize the overlap, we adopted a different color-coding strategy than the one used for overlaying multiple populations in the same anatomical space. For each population, we used three colors to denote the three possible states when overlaying two modalities: green represented overlap, red represented space occupied exclusively by a population and orange represented space occupied exclusively by LRPNs classified as members of the population. The same color-coding was used for the sub-cortical visualizations. We then stacked all generated flatmaps together to assess the overlap across all populations (Fig. 8A-G). To summarize the flatmap overlays, we visualized for each population the ratio of overlap or dominance by the population and proximal LRPN neurons across the primary and supplemental somatosensory areas (see Fig. 8I-J). For each target area, we plotted only the axonal overlap of populations who sent the majority of their axons to that area, in order to highlight the projection target-specificity of each population alongside its agreement with the LRPN morphologies or AMBCA experiments.

We found a substantial overlap between the morphology and population cortical target surfaces for the population group that most dominantly targeted SSp (Fig. 8A-D): SSp-m (experiment 1), SSp-n (exp. 4) and SSp-bfd (experiments 2 and 3, with exp. 2 targeting SSp-bfd exclusively and exp. 3 also jointly targeting SSp-bfd and SSs). In this first group, we observed specificity of population target surface, meaning that the targeted cortical surface of populations substantially overlapped with that of morphologies (45% for exp. 1, 27% for exp. 4, 20% and 14% for experiments 2 and 3, respectively).

We have not yet found morphologies whose somata fell into the injection volume site of a population, which means that there are potential topographical differences in their targets. The use of a relaxed radius to find the nearest neurons was thus a necessity for having an adequate sample size of morphologies to compare with the populations (see Section S1.6). However, this resulted in stricter topographical specificity of populations compared to their most proximal neurons. That could partially explain the substantial targeting of cortical surface by morphologies that was exclusive instead of overlapping with the populations. An example case is experiment 2, whose most proximal neurons were distributed all over the whisker representation in VPM, while the injection volume of exp. 2 only labeled a fraction of it (see Fig. S1). This difference in localization specificity was reflected in the cortical surface, since experiment 2 specifically targeted the anterior-lateral part of the barrel cortex, while the most proximal neurons jointly targeted a larger fraction of the barrel cortex.

An additional reason was the under-representation of certain subsets of VPM neurons. For instance, experiments 3-4 are populations in VPMvl and as such are not properly represented in the single-neuron experiments. This lack of VPMvl neurons caused the selection of morphologies whose somata do not reside in VPMvl and thus had different projection patterns than experiments 3-4.

In the second population group that most dominantly targeted SSs, we observed a weaker overlap, with the exception of experiment 5 (41% for exp. 5 compared to 9% and 15% for experiments 6 and 7, respectively, see Fig. 8E-G). For experiment 5, we observe specificity of morphological target surface, with the targeted cortical surface of morphologies strongly overlapping with populations, but the reverse is not true. Similarly to VPMvl, an explanation could be given by the available samples from the Mouselight and Braintell databases. Despite the two databases together having provided an adequate sample size of 257 morphologies which strongly target SSp, SSs is underrepresented. Therefore, the presence of a minority morphological group highly overlapping with a population in targeted SSs surface may allude to the presence of a new projection type that previous works have not properly characterized.

### Micropopulation Experiments have a Higher Topographical Precision than Proximal Experiments from the AMBCA

Lastly, we integrated the population data with the most proximal tract-tracing experiments from the AMBCA dataset (Oh et al., 2014; Harris et al., 2019), based on their injection volume locations in a similar fashion to the single-neuron experiments (see "Integrating Additional Modalities"). Both modalities were visually overlaid using cortical flatmaps and VPM maximum projection plots similarly to "Single Neuron Projections Match Population Tracing Data for the SSp-Targeting Experiments". The motivation was to assess the topographical specificity of BDA tracers compared to AAV tracers. The IDs of the matching AMBCA experiments that were proximal to our seven experiments were: 268206050 for exp. 1, 478581080 for exp. 2, 158375425 for exp. 3, 158375425 for exp. 4, 268399868 for exp. 5, 268399868 for exp. 6 and 268399868 for exp. 7.

**Fig. 9:**
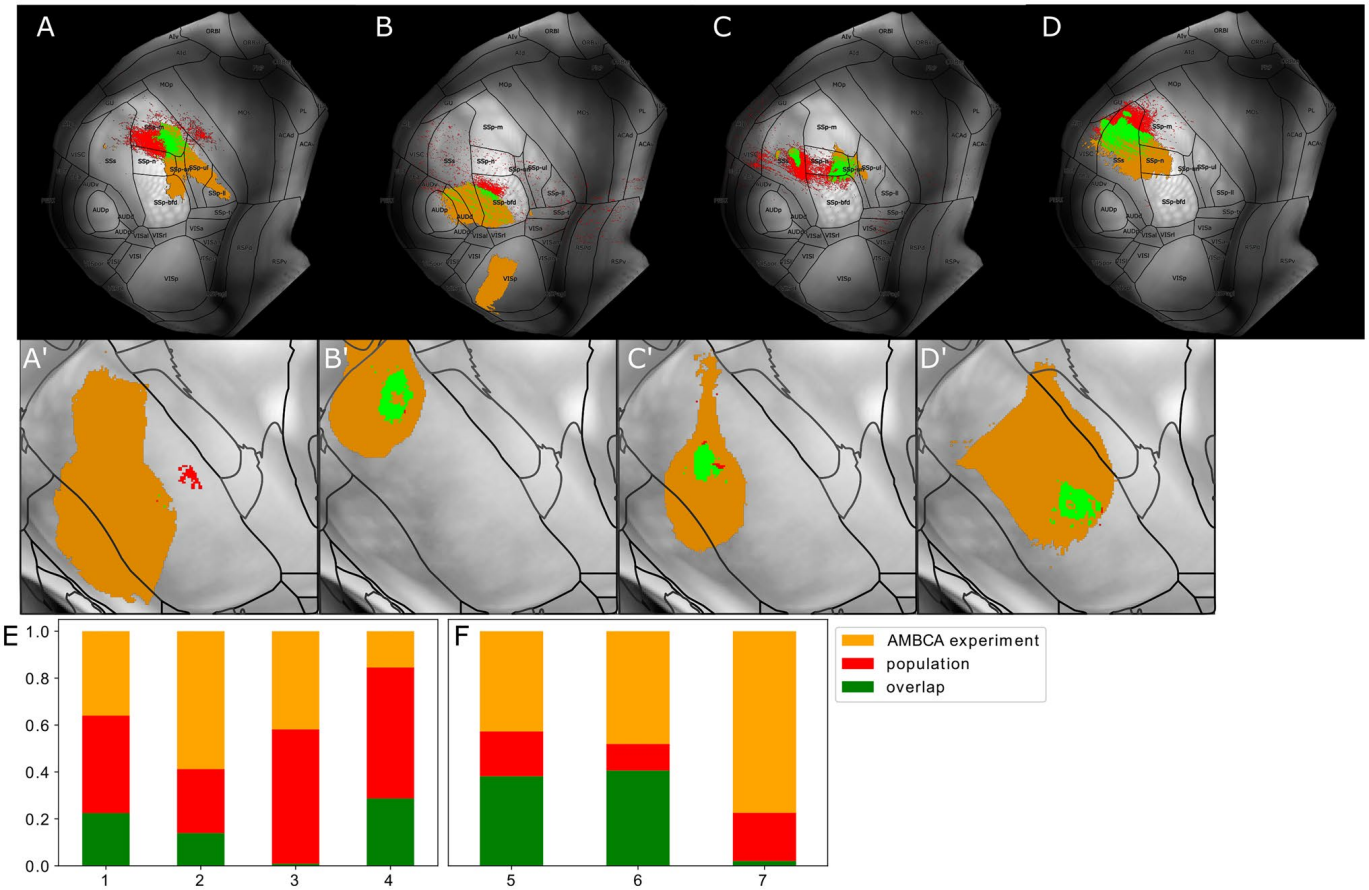
Spatial overlay of registered population experiments with anterograde tract-tracing experiments from the Allen Mouse Brain Connectivity Atlas (AMBCA), which were labeled with the AAV virus instead of BDA, demonstrates the finer topographical precision of the former compared to the latter. Experiments 1-7 correspond to experiments **A**-**G** from Figs. 7-8. **A**-**D** Dorsal cortical flatmap representation of the axonal segments of a population, one per plot, overlaid with a proximal AMBCA population. The plots are similar to those in Fig. 8. **A’**-**D’**. Maximum projection plots of VPM across the coronal plane (10 $\mu$m) that illustrate the overlap of the above mentioned populations at the level of their injection volume. The gray colour corresponds to the background gray-matter volume from the STP images of the CCF v3.0 and the black contour lines delineate the anatomical borders of VPM according to the ARA parcellation. **E**-**F** Fraction of points in flatmap space targeted by a population, AMBCA experiment or both within SSp (**E**) and SSs (**F**), across the different populations. x-label: letter denoting the experiment id described above. y-label: percentage of targeted points that belong to each of the three states, which is normalized by all points within SSp (**E**) or SSs (**F**). For each somatosensory area, we plot only the populations that most dominantly target the area. The color-coding is consistent with the previous plots. Experiments 3, 6 and 7 were excluded from the figure since they matched already shown AMBCA experiments, specifically: experiment 3 matches the experiment shown in **D**, while experiments 6 and 7 match the experiment shown in **E**

The populations corresponding to the AMBCA experiments (see Fig. 9), with the exception of the one that was proximal to experiment 2, covered topographically large parts of VPM spanning dorsal-mid-ventral positions. This was reflected in the multi-target projections of the AMBCA populations that simultaneously covered a large fraction of the somatosensory cortex. For instance, the AMBCA populations being proximal to experiments 4-5 targeted both SSp and SSs and the population proximal to exp. 1 targeted multiple areas in SSp. Moreover, the population proximal to exp. 2 targeted the visual cortex due to the injection volume spreading over to the lateral geniculate nucleus (LGN). This was further corroborated by the fact that all four AMBCA injection volumes shown in Fig. 9 spread over to neighboring nuclei beyond VPM.

With the exception of the population proximal to exp. 1, we noticed that the injection volumes of our populations were a fraction of their respective matching AMBCA injection volumes. Furthermore, the majority of the AMBCA populations had a higher coverage of cortical surface compared to our populations in terms of axonal segments (see Table 2). The only exception in cortical surface coverage was for the populations proximal to experiments 3-4, since these experiments were derived from cre-lines and had a more restricted injection volume in the mid-lateral part of the dorsal-ventral plane of VPM.

Given the topographical precision of the injection volumes of our populations, as well as the matching of multiple of our populations to the same AMBCA population and their respective difference in magnitude of projections, we deduce that BDA is a more appropriate tracer for studying the topographical organization of VPM and potentially of other nuclei of a similar volume and size (Wang et al., 2014).

**Table 2:** Fraction of coverage of cortical surface by the populations that have been registered using this pipeline (columns 1 and 2), in comparison to their matched experiments from AMBCA (columns 3 and 4)

	Population cortical coverage	AMBCA experiment	AMBCA cortical coverage
Exp. 1	1.3%	268206050	1.8%
Exp. 2	1.4%	478581080	2.3%
Exp. 3	1.25%	158375425	0.7%
Exp. 4	1.0%	158375425	2.3%
Exp. 5	1.5%	268399868	0.7%
Exp. 6	0.8%	268399868	2.6%
Exp. 7	0.5%	268399868	2.3%

Taken together, this pipeline offers the possibility of additive integration of data from different institutions and research groups to create a comprehensive repository of mesoscale connection data that could be used for the biologically accurate modelling of forebrain networks.

## Discussion

In this work, we have developed a pipeline for registering, integrating and analysing multiple tract-tracing experiments together in the Allen Common Coordinate Framework. The pipeline can be seen as an alternative to the QUINT workflow, with the key distinguishing feature being the inversion of the registration step of QUINT: the experimental data are non-linearly registered to match the anatomical template, which is a necessary pre-requisite for integrating multiple experimental data to the same coordinate space. To be capable of targeting topographical subdivisions of an area under study, we use an anterograde BDA tracer which labels 10-100 cells yielding a high spatial precision compared to other bulk labeling approaches. For instance, the recombinant adeno-associated virus (AAV), which has been used in large scale datasets (Oh et al., 2014; Harris et al., 2019), can simultaneously label hundreds of cells, thus hindering the study of subdivisions in smaller brain areas or nuclei. To illustrate the effectiveness of the pipeline, we have focused on registering seven anatomically distinct populations of VPM neurons to the CCF. VPM was selected as a simple test case in topography due to its lower morphological diversity as a lower order thalamic nucleus compared to higher order nuclei. The populations were registered using a sequential application of an image pre-processing procedure, followed by the well-established DeepSlice, QuickNII, VisuAlign and ilastik tools of the QUINT workflow, followed by the OpenCV library for post-processing (Bradski, 2000). The laminar, projection and topographical patterns of these populations were visually inspected with the use of cortical flatmap and subcortical visualizations. To cross-reference the results with previous findings, we spatially overlaid the projections of populations at the cortical surface with projections from anatomically proximal single-neuron morphologies obtained from the Mouselight and Braintell databases and from alternative tract-tracing experiments in the AMBCA dataset.

We identified a topographical organization of VPM neurons varying across the dorsal-ventral axis projecting progressively to the barrel cortex, nose and mouth, and varying across the anterior-posterior axis projecting progressively from the primary to the supplemental somatosensory area. This is in line with findings in mouse (Peng et al., 2021) and rat somatotopy (Waite, 1973; Saporta & Kruger, 1977; Ito, 1988; Sugitani et al., 1990), which have all reported a topographical correspondence between the location of the VPM soma and its respective centroid of cortical axonal terminals in terms of 3D rotation from the ventral-dorsal and medial-lateral axes to the anterior-posterior and lateral-medial axes. Moreover, we found that the registered populations could be characterized by distinct projection patterns in the somatosensory cortex, specifically to the primary barrel field, mouth and nose, as well as to the supplemental somatosensory area.

When comparing our populations to proximal populations from the AMBCA, we observed a finer topographical targeting both at the level of injection volumes, which are topographically precise while AMBCA volumes spread to surrounding nuclei, and at the level of cortical targets, which cover smaller surface and target fewer areas compared to AMBCA. The large size of transfected thalamocortical cell populations from AMBCA was, in fact, one of the main limitations found by Knox et al. (2018) when they set out to build a model of the mouse connectome based on these datasets (Knox et al., 2018).

The primary projection patterns were validated because they overlapped with the single-neuron projections on the cortical surface. However, we did not find a substantial overlap between the projections of single neurons and our micropopulation experiments in SSs. The existence of denser axonal segments from our populations compared to the single-neuron reconstructions could suggest that some VPM neuron subpopulations may not be included in the single-cell databases. That said, results are currently considered inconclusive and will be further investigated when the full data set has been processed and analysed. This is a pilot study of this system using the pipeline, and a much larger number of micropopulation experiments should be added to saturate the diversity/variation of the nucleus neurons and their cortical connections.

Mismatches between the two modalities could be attributed to several problems related to single-neuron reconstructions. First, there have been reports of incomplete morphologies in studies that utilize this approach (Liu et al., 2022), something that was foreshadowed by the lack of the thalamo-reticular branch in many publicly-available reconstructed morphologies, a feature considered to be universal for all thalamic neurons (Jones, 2007; Clascá, 2022). Secondly, registration errors, however small, can add significant noise to the subsequent analysis. Lastly, the use of Cre-dependent lines and vectors could be providing a sample of thalamic cell types that is skewed or incomplete. For example, in the current Braintell dataset (as of June 2023), for example, the cells of the first-order nuclei of the thalamus are clearly over-represented, and within the VPM dataset, virtually all the neurons seem to be of the classic mono-focal type (Clascá, 2022) and thus part of the lemniscal pathway, with no representatives of the multi-focal morphology that characterizes the extralemniscal VPM pathway (Pierret et al., 2000; Zhang et al., 2021).

Regarding laminar specificity of projections, layer 4 was strongly targeted by all populations. Moreover, populations branching to multiple targets had dense arborizations in layer 2/3. These populations are located at the ventrolateral part of VPM and play a distinct functional role as a relay for the extralemniscal pathway (Pierret et al., 2000; Yu et al., 2006; Zhang et al., 2021). Layers 5 and 6a were also strongly targeted by multiple populations, but this is artifactual as it was due to the presence of corticothalamic somata and dendrites that were retrogradely labeled by the experimental labeling procedure.

To our knowledge, this is the first open-source pipeline that combines registration, segmentation, integration and analysis of multiple tract-tracing experiments, as well as anatomical overlay with different reference atlases and previously-registered tract-tracing experiments and single-neuron reconstructions. It is important that mention that the individual components of the pipeline that are shared with the QUINT workflow have not been improved qualitatively compared to the latter. Therefore, the innovation of this pipeline lies in the novel fashion that its individual components and modules have been re-used, modified and integrated to provide results such as the ones demonstrated in this work. The most markedly significant modification was reversing the non-linear registration performed by VisuAlign, which enabled the registration of experimental sections to a common reference space and the consequent integration of multiple registered datasets to this shared space. This allowed us to compare the registration results to the gold standard datasets of AMBCA, Mouselight and Braintell and find additional structure that was not present in the latter ones. Use-cases are not limited to tract-tracing experiments but they can be extended to include multiple section imaging modalities of the adult mouse brain, which are more cost effective compared to 3D data modalities.

This bringing together of data registration, fusion and analysis is thus the main distinction of this pipeline from previous workflows and tools, such as QUINT or the Software Development Kit (SDK) of the Allen Institute (see Table 1). QUINT focuses on the steps leading to the registration of a neuroanatomical dataset and the subsequent analysis of it in isolation from other data sets. Allen SDK provides tools for the analysis of multiple datasets, which have to be already been registered. Moreover, the interpretation of the results is enhanced in our pipeline by extensive visualization options in the form of cortical flatmaps (Knox et al., 2018), 2D subcortical projection plots and 3D rendering by the SBA composer (Bakker et al., 2015).

An additional strength of this pipeline compared to previous tools is that it is comprised of a Jupyter Notebook that does not require detailed knowledge of programming. Its compatibility with the QUINT workflow enables the additional analysis of previously registered datasets using our methods. Instead of being restricted to the ARA parcellation, the pipeline can be extended to include additional anatomical parcellations for visualization and analysis such as the EUAL. These integrative features of the pipeline can lead to the testing of hypotheses regarding the somatotopical organization of thalamic nuclei, which we will elaborate on in the following paragraphs.

That said, we acknowledge a number of limitations of the pipeline. A major current limitation is that the segmentation of neurites by ilastik can be highly sensitive to the contrast of the training images. Different immunohistochemical methods produce different tissue staining, which will result in images with different contrast between signal and background. Hence, besides errors caused by the experimental procedure, false positives can also be attributed to the ilastik classifier. A challenge is presented by the lack of ground truth labels to quantify the classification accuracy or rather the false positive rate, which in this case is the most important criterion for the segmentation quality. Therefore, the most viable criterion is the evaluation of the segmentation by an expert neuroanatomist. As we showed in "Step-by-Step Description of the Pipeline", false positives can also be eliminated if we know a priori that a signal in some areas is almost certainly erroneous: in our case, the presence of detected soma volume outside of VPM, which is not possible if the neuroanatomist has deliberately targeted that area. We intend to improve the image segmentation by training different classifiers for different types of staining and then asking the user to specify the applied staining procedure in the file name descriptor of each image. A Python script would then be used to apply the correct classifier to each image by reading its file name descriptor.

An additional limitation is the presence of a discontinuity in the pipeline, since not all steps can be performed serially with the execution of the Jupyter Notebook. In particular, two registration steps require the execution of QuickNII and VisuAlign. This can be seen as a bottleneck in the pipeline’s smooth iteration: the user needs to pause using the Notebook after the DeepSlice application step to execute the QuickNII and VisuAlign software packages separately, and then resume the Notebook for the image segmentation and reverse registration steps. This bottleneck will be overcome in the version of the pipeline that will be uploaded at the EBRAINS Collaboratory (see Table 1). In the collaboratory, an API interface will establish real-time communication between the currently running Jupyter Notebook and the web versions of QuickNII and VisuAlign, namely WebAlign and WebWarp, respectively. The API will allow the user to directly navigate to WebAlign with a pre-loaded image section for registration, thus allowing a fast and efficient linear correction of the DeepSlice registration. The user can then subsequently navigate to WebWarp with the pre-loaded anchor points from WebAlign, where they can make the non-linear refinements to the registration. The results obtained from WebWarp will be directly loaded to the Jupyter Notebook, hence proceeding with the next steps without any disruption of the pipeline’s flow.

The last limitations to acknowledge are related to the sparsity of registered tracing data and the presence of retrograde signal in the data. The sparsity was caused by inter-spacing gaps from the tissue sections across the anterior-posterior axis, which resulted in missing neurites. To mitigate this issue, we will implement a spatial interpolation approach for imputing the missing segments between two consecutive tissue sections (Meijering et al., 2001). The retrograde signal is illustrated in Fig. 7I-J, which display a higher volume of axonal arborizations in layers 5 and 6 than expected. This was due to the accidental retrograde labeling of corticothalamic somata, which has been previously reported in anterograde tract-tracing experiments with BDA-tracers (Wang et al., 2014).

Regarding future extensions to the pipeline, our primary goal is to build a connectivity matrix of VPM and potentially other thalamic nuclei, such as the Posterior nucleus (PO), which would be a significant improvement on the classical connectivity matrix provided by (Oh et al., 2014; Knox et al., 2018). In the previous matrices, connections are defined by the projection densities connecting distinct brain areas. Despite their significance in understanding rodent brain connectivity, these matrices do not take heterogeneity of projections into account, which, as we saw in this work, is present even in simple nuclei such as VPM. The new connectivity matrix should in principle be a somatotopic model for VPM, in which one could traverse the voxels comprising VPM and retrieve for each one of them the most densely targeted cortical subvolume of the common reference space. Given the size of VPM and our injection experiments, we estimate that the number of populations required for building such a matrix could be in the range of 50-100, provided that they are evenly distributed across the nucleus.

Taken together, in this work we have provided the basis towards a somatotopic subdivision of VPM based on its projections to the different territories of SSp and SSs. This was achieved by meeting two conditions, namely the use of BDA tracers for topographically-precise micropopulation labeling, and a publicly accessible pipeline for registration of the resulting section images to CCF and their subsequent visualization, analysis and validation with other modalities. The registration to a common reference space allowed us to incorporate a dataset, comprised of multiple topographically organized projection patterns from VPM, into a virtual atlas that is to be shared, expanded and improved upon by the neuroscience community. This can set the basis for multimodal data integration into a whole-brain connectivity matrix that can potentially correlate anatomical and physiological data. In the following paragraphs, we will discuss the feasibility of this aim.

It is first important to state that the release of a standardized pipeline for the accurate registration of section imaging data to the Allen CCF v3.0, which is currently the gold standard reference template, is a prerequisite for promoting open science. It ensures the maximal integration and reproduction of experiments from smaller scale laboratories that do not have the necessary resources for expensive in-house image registration workflows. Releasing easily accessible and publicly available registration pipelines can incentivise their application to a larger number of laboratories, which in turn can increase the number of registered experiments, which in turn can increase the amount of publicly available and reusable data.

Besides reproducibility, it is even more important if such registered datasets can provide the resource to answer multiple questions (Abbott et al., 2020). As we have seen in numerous works that inspired us (Oh et al., 2014; Zingg et al., 2014; Jeong et al., 2016; Bienkowski et al., 2018; Harris et al., 2019), registering data obtained from multiple tracing experiments to the same reference space allows us to perform spatial statistical analyses for uncovering the underlying variability in the topographic relationship between the location of somata and their axonal projection patterns across the anatomical sub-divisions of the brain. This would be impossible to do if common spatial coordinates did not exist. Anatomical subdivisions based on topographically distinct projection patterns have been shown to be a robust measure for refining the anatomical labeling and parcellation of the brain, regardless of the utilized parcellation scheme (Chon et al., 2019). Sharing such open source data can only further improve the thalamic refinement and make progress towards a common parcellation system besides a common reference space.

While mouse thalamocortical tract-tracing experiments were the major focus of this work, there is a plethora of other data modalities that could be immediately registered with this pipeline without further tweaks. It is commonly accepted that multi-modal data integration over the same spatial location can substantially increase their value (Timonidis & Tiesinga, 2021). As the pipeline shares with QUINT the software used for the registration, previously QUINT-registered datasets can also be further processed and analysed by our methods. The first question is which other species could be registered. The pipeline requires the availability of at least one anatomical template and reference atlas for a given species. A prime example is the rat for which the Waxholm Space Atlas of the Sprague Dawley Rat Brain (WHS Rat) can be used (Papp et al., 2014).

For a species that satisfies this criterion, any modality that can be obtained in the form of 2D section images can be integrated. Exemplar modalities for integration in the pipeline are densities of cell bodies (Kim et al., 2017) or synapses (Zhu et al., 2018), as well as In Situ Hybridization-based spatial transcriptomics (Lein et al., 2007). For instance, overlaying the spatial distribution of axonal projections from VPM to the mouse barrel cortex with the distribution of cell densities of barrel-specific inhibitory and excitatory populations, can provide insight on the functional role of each projection, which would not be possible by examining each modality alone. This can lead to developing a direct cell-type-specific connectivity matrix, which is of particular importance to brain network models since the inhibition-to-excitation ratio of different cell-types within a cortical column can now be constrained by actual biological data instead of by proxy measures, such as the exponential distance rule or statistics derived from previous experimental works (Hua et al., 2022).

To take one step further into understanding brain function however, one would need to put physiology into the context of anatomy. This would necessitate the spatial registration of experiments from modalities such as patch-clamp recordings (Hill & Stephens, 2021), calcium imaging (Grienberger & Konnerth, 2012) or extracellular recordings with multi-site probes (Buzsáki et al., 2012). As a use case for VPM, somatotopic thalamocortical connections could be investigated for the potential generation of alpha-like rhythms in awake mice, which has been hypothesized to involve thalamic bursting through low-threshold calcium spikes (Sobolewski et al., 2011).

Lastly, computational neuroscientists can benefit from connectivity produced by our pipeline by implementing biologically realistic rules for placing and connecting cells in simulated thalamic and cortical-column circuits, which would be based on the topographic organization observed in the matrix. Following this plausible configuration, the modeler could then initiate a simulation of network activity with the use of spiking neural networks, such as the Potjans-Diesmann model (Potjans & Diesmann, 2012).

## Information Sharing Statement

The pipeline described in this work has been designed and tested in the form of a Jupyter Notebook together with a number of supporting libraries/modules in Python. All respective code has been published online with supporting description and documentation at the EBRAINS Collaboratory and at Github in the form of a neuroinformatics-related tool, namely *population-integrator*. See Main Table 1 for links to the public repositories of the tool and modules mentioned here.

### Supplementary Information

Below is the link to the electronic supplementary material.

Supplementary file4 (PDF 173 KB)

## Data Availability

The segmented section images, the registration output files and the analysis results that support the findings of this study are available at the EBRAINS Collaboratory and at Github together with their corresponding Python/Jupyter Notebook code, as described at the Information Sharing Statement. The original Minimum-intensity projection section images that constitute the raw input of our pipeline are currently in the process of being curated and published in EBRAINS according to the FAIR Guiding Principles. In the meantime, the dataset is located in controlled access data storage at Radboud University Nijmegen/Autonoma de Madrid University and it can be available from the corresponding author upon request.
