# Supplementary material for: Analyzing Thalamocortical Tract-Tracing Experiments in a Common Reference Space
Source: Neuroinformatics. 2023 Oct 21;22(1):23–43. doi: 10.1007/s12021-023-09644-4 (PMC10917831; doi:10.1007/s12021-023-09644-4)
Supplement: Supplementary file 1 — Supplementary file4 (PDF 173 KB) [file 12021_2023_9644_MOESM1_ESM.pdf]

Neuroinformatics manuscript No.
(will be inserted by the editor)

Analyzing thalamocortical tract-tracing experiments in a common reference space

Nestor Timonidis[1,*] · **Mario Rubio-Teves**[2,*] · **Carmen Alonso-Martínez**[2] · **Rembrandt Bakker**[1,3] · **Maria Garcia-Amado**[2] · **Paul Tiesinga**[1,+] · **Francisco Clascá**[2,+]

Received: date / Accepted: date

Nestor Timonidis
[1]Neuroinformatics department, Donders Centre for Neuroscience, Radboud University Nijmegen, Heyendaalseweg 135, 6525 AJ Nijmegen, the Netherlands
Tel.: +31-649552777
E-mail: nestor.timonidis@donders.ru.nl

Mario Rubio-Teves
[2]Department of Anatomy and Neuroscience, School of Medicine, Autónoma de Madrid University, C. Arzobispo Morcillo 4, 28029 Madrid, Spain
Tel.: +31-649552777
E-mail: mariorubioteves@gmail.com

Carmen Alonso-Martínez
[2]Department of Anatomy and Neuroscience, School of Medicine, Autónoma de Madrid University, C. Arzobispo Morcillo 4, 28029 Madrid, Spain

Rembrandt Bakker
[1]Neuroinformatics department, Donders Centre for Neuroscience, Radboud University Nijmegen, Heyendaalseweg 135, 6525 AJ Nijmegen, the Netherlands
[3]Inst. of Neuroscience and Medicine (INM-6) and Inst. for Advanced Simulation (IAS-6) and JARA BRAIN Inst. I, Jülich Research Centre, Wilhelm-Johnen-Strasse, 52425 Jülich, Germany.

Maria Garcia-Amado
[2]Department of Anatomy and Neuroscience, School of Medicine, Autónoma de Madrid University, C. Arzobispo Morcillo 4, 28029 Madrid, Spain

Paul Tiesinga
[1]Neuroinformatics department, Donders Centre for Neuroscience, Radboud University Nijmegen, Heyendaalseweg 135, 6525 AJ Nijmegen, the Netherlands

Francisco Clascá
[2]Department of Anatomy and Neuroscience, School of Medicine, Autónoma de Madrid University, C. Arzobispo Morcillo 4, 28029 Madrid, Spain

* These authors contributed equally
· + These authors contributed equally

1 Supplementary Materials

1.1 AMBCA

The *Allen Mouse Brain Connectivity Atlas* (AMBCA) repository offers the largest collection of anterograde tract-tracing experiments spanning the entire mouse brain to date (see 1). It is an ensemble of $\sim$ 1600 experiments performed on 56-days-old (P56) wild-type (C57BL/6J) and transgenic cre-line male mice, with 16 different driver lines in total (Oh et al., 2014; Harris et al., 2019). They used a recombinant adeno-associated virus (rAAV) expressing enhanced green fluorescent protein (EGFP) to label the wild-type mice (Oh et al., 2014). To label the transgenic cre-line mice, they used a cre-recombinase-dependent rAAV virus expressing either a EGFP expressed in the neuronal cytoplasm or a fusion of synaptophysin-EGFP that labels the pre-synaptic terminals (sypEGFP) (Harris et al., 2018). The main difference is that in the wild-type experiments, most neurons at the injection location were labeled, whereas in the cre-line experiments, only neurons belonging to specific cell-classes were labeled. They visualized the labeled axonal projections using two-photon microscopy and produced brain slice images at 1 μm resolution, which have been registered to CCF v3.0 (Wang et al., 2020), thus producing a whole-brain volumetric dataset.

A number of cre-line experiments targeted the projections of multiple thalamic nuclei which were thus classified into different projection classes based on the cortical hierarchy of their projections as feedforward or feedback (Harris et al., 2019), In particular, nuclei were classified as core (predominantly targeting cortical areas in layer 4), intralaminar (predominantly targeting the striatum), matrix focal (predominantly targeting layer 1 of a single area) and matrix multi-areal (predominantly targeting layer 1 of multiple areas).

That said, the coarseness of the delineation of mouse brain areas based on their projection profile is due to the technical constraints of the experiments performed for AMBCA. A major limitation is the dense labeling induced by the selected viral tracers, which resulted in an inability to further subdivide brain areas and nuclei by topographically organized neuronal sub-populations with different projection patterns. Likewise, it is not possible to distinguish the precise termination patterns of different classes of cells labeled by the cre-line tracers. While this issue has been largely mitigated by the emergence of large-scale datasets of axonal morphological reconstructions of single-neurons (Winnubst et al., 2019; Peng et al., 2021), thousands of similar reconstructions will be required to reach a coverage of thalamic nuclei similar to (Harris et al., 2019).

1.2 QUINT workflow

The QUINT workflow is comprised of three well-established neuroinformatics tools, with each tool corresponding to a step of the workflow. First, the QuickNII software (Puchades et al., 2019) is used to register experimental section images to the CCF template, followed by generating images of the corresponding template section. Second, the ilastik software (Berg et al., 2019) is used to segment useful objects out of

the experimental section, such as cell bodies or neurites. Third, the Nutil software (Groeneboom et al., 2020) is used to quantify the counts of the segmented objects from step 2 across the delineated brain regions that characterize the template section from step 1. In the following paragraphs, we will provide a short description of the QuickNII and ilastik tools. We will also describe two additional tools related to QUINT that were incorporated in our pipeline, namely VisuAlign (see 1) and DeepSlice (Carey et al., 2022), which corroborated the registration procedure. Lastly, we did not use the Nutil software but utilized the Python-based libraries NumPy and Matplotlib to compute counts of segmented pixels directly within the resulting 3D volume (see 1) that was generated by the finalized registration of a complete experimental dataset.

1.2.1 QuickNII

QuickNII is a semi-automated open-source software that assigns a spatial location to serial section images. In particular, it performs affine spatial registration of sectional image data to a 3D reference atlas coordinate framework. It enables the manual selection of the best fitting atlas slice for a given experimental slice through the visually maximal overlap of both slice images.

QuickNII produces customised atlas maps adapted to match the cutting plane and proportions of the sections. The location is defined by superimposing the reference atlas onto the section images (anchoring). The cutting angle of the reference atlas is adjusted to match the section plane identified using an affine transformation, which is followed by a manual adaptation of the atlas section to match the experimental section.

Then, the transformation of pixel coordinates to the corresponding voxel coordinates in the atlas, generated by QuickNII, is given by:

$$\begin{pmatrix} x_v \\ y_v \\ z_v \end{pmatrix} = \begin{pmatrix} u_x & u_y & u_z \\ v_x & v_y & v_z \\ o_x & o_y & o_z \end{pmatrix} \begin{pmatrix} 1/w & 0 & 0 \\ 0 & 1/h & 0 \\ 0 & 0 & 1 \end{pmatrix} \begin{pmatrix} x_p \\ y_p \\ 1 \end{pmatrix}, \quad (1)$$

where x_v,y_v,z_v are 3D voxel coordinates in atlas space, x_p,y_p are 2D pixel coordinates of the experimental slice, u,v,o correspond to the top right, bottom left and top left corners of the image in the atlas space, respectively, and w, h correspond to the width and height of the registered image.

In the case of non-linear refinements applied on top of the linear registration, the experimental slice pixel coordinates are non-linearly transformed to match the pixel coordinates of the corresponding atlas slice ($\hat{x}_p$,$\hat{y}_p$) (see section S1.2.3) and the above equation is adapted to work with the transformed coordinates instead.

1.2.2 ilastik

Ilastik is a user-friendly tool that performs image analysis using machine learning methods without requiring methodological expertise from the user. It solves multiple image analysis problems with the use of supervised classification algorithms. Classification focuses on training models to learn how to classify data into two or multiple

categories, by providing them exemplar input data that have been a priori associated with certain categories, followed by testing the performance of the model on previously unseen examples (Tan et al., 2005; Bishop, 2006) One of the major workflows of ilastik is the pixel classification workflow, which aims to produce a characterization of pixels in an input image that is of significance to the user. This workflow segments images in a semantic fashion: it associates a label to each image pixel, which is defined by the user using a graphical user interface. Given semantic classes to which the labels refer, the user has to provide examples within the image that correspond to the different classes, by painting each example with a graphical brush using a colour that is associated with the class. Ilastik then estimates the probability that each pixel belong to a class. In terms of technical implementation, the input of this workflow is the output of a number of filters applied to the image, which have also been selected by the user. The method used to train the classifier is typically the Random Forest Classifier comprised of 100 decision trees (Breiman, 2001), motivated by the low number of required parameters and its high performance in image classification tasks (Tan et al., 2005).

1.2.3 VisuAlign and reverse registration

VisuAlign is a sibling tool to QuickNii that utilizes a user interface for refining an already existing 2D affine registration in a non-linear fashion. It has been specifically developed for curating the spatial variability resulting from a linear registration of an experimental section image to an atlas-derived one. VisuAlign thus employs user-generated landmarks in the user interface to perform a non-linear transformation or ”warping” of the atlas slice to the experimental one.

To understand how the reverse non-linear registration of an experimental section to an atlas section was achieved, it is first important to clarify the implementation of the forward registration. For that purpose, let us label the pixel coordinates of the experimental section as the ”to” coordinates, and the corresponding atlas section ones as the ”from” coordinates. The forward registration has been selected as the default option of registration in Visualign’s user interface, because it eliminates the issue of folding: a single point in the ”from” can transform into multiple ”to”.

VisuAlign utilizes the Delaunay triangulation technique, which is implemented by the incremental Bowyer-Watson algorithm (Bowyer, 1981; Watson, 1981). First, a rectangle is used to represent the original pixel coordinate space, on which the experimental and atlas sections are overlaid. The rectangle is defined by the width and height of the experimental section:

$$\begin{aligned} P0 &= [-0.1*w, -0.1*h], \\ P1 &= [1.1*w, -0.1*h], \\ P2 &= [-0.1*w, 1.1*h], \\ P3 &= [1.1*w, 1.1*h], \end{aligned} \tag{2}$$

where w and h correspond to the width and height of the pixel coordinate space. The reason why the rectangle occupies a 10% larger space that than the original pixel space is to allow for a higher range of overlay between the two sections. For

the triangulation to take place, the rectangle has to be partitioned into two triangles that are formed by [P0, P1, P2] and [P1, P2, P3], respectively. The formation of the two triangles now allows each pixel inside the image to be represented via barycentric coordinates (λ_1, λ_2, λ_3), which can return the original pixel coordinates if the dot product is taken with respect to the triangle vertices. This barycentric coordinate representation results in a computationally efficient mapping between the ”to” and ”from” coordinate systems.

The forward transformation is thus achieved by estimating for each pixel the respective ”from” coordinate, followed by transforming the ”from” coordinates to the ”to” coordinates in their barycentric form. As soon as the forward mapping has been completed, the reverse transformation from a ”to” to a ”from” point can be achieved in the reverse fashion.

1.2.4 DeepSlice

DeepSlice is a deep neural network that has been trained on thousands of histological images to automatically register coronal section images to the Allen Mouse Brain CCF v3.0 (Carey et al., 2022). It uses the Xception architecture front-end (Chollet, 2017), which performed well on classifying images utilizing a deep architecture of convolutional layers, combined with a new back end that predicts where the corners of slice are placed within the Allen Mouse Brain Atlas (origin, left edge, bottom edge). This model was trained using a variety of brain section images from the Allen CCF v3.0 with their position as determined by the Allen image registration pipeline (see 1), as well as synthetic slices generated by Nissl and serial two-photon image volumes from the Allen Institute. The performance is improved when the slice index (from experiment) and cutting angle is integrated across the slices.

1.3 Flatmap creation

To provide a 2D visualization of neuroanatomical information within the spatial context of the cortex, in the form of a flatmap, the following two-step procedure was followed (Harris et al., 2019). First, a curved cortical coordinate system was defined, followed by a smooth mapping of the cortical surface to a flatmap. For the first step, the orthogonal path between the pia surface and the white matter was computed with the use of the Laplace equation (Griffiths, 2013). This resulted in the creation of streamlines as the third axis in the cortical surface, which match columnar cortical structures and provide information about cortical depth. The streamlines could thus be averaged over the desired range of cortical depth which then leads to a 2D embedding of the data. For the second step of the flatmap creation, the geodesic distance was computed from each point of the surface to pairs of anchor points that formed the two respective axes of the 2D embedding into a flatmap. The position of each cortical source point into the flatmap was inferred by finding the flatmap point at which the radial distance from the anchor points in 2D equals the geodesic distance between the source point and the anchors in 3D.

1.4 EUAL parcellation

The Enhanced and Unified Anatomical labeling (EUAL) parcellation is a unified anatomical labeling system which integrates the labeling of the Franklin-Paxinos (FP) atlas (Paxinos and Franklin, 2019) into the Allen CCF v3.0 (Chon et al., 2019). The aim of this system is to minimize discrepancies in the anatomical delineations of the mouse brain between the two predominant atlases, namely FP and ARA. The integration was achieved by first identifying and aligning corresponding points between the FP and ARA labels. They then used spatial variations in volumetric cell density data, obtained from transgenic cre-line mice (Kim et al., 2017), as a proxy for further subdividing ARA labels whose corresponding FP labels have a higher degree of subdivision. In addition, they validated the parcellation by performing a topographical analysis of connectivity patterns obtained from tract-tracing data (Oh et al., 2014; Hintiryan et al., 2016; Harris et al., 2019).

1.5 SBA Composer

The Scalable Brain Atlas (SBA) Composer is a web-based application for displaying brain imaging, volumetric and 3d-object-based data embedded within a number of available brain atlases (Bakker et al., 2015). It can be used directly by users or by third party websites as a visualization front-end (see Main table 1 for more details). It uses the anatomical parcellation of the Allen Brain Atlas, the areas of which can be rendered alongside the imported data with parameters for color and transparency that can be selected by the user. Moreover, the user can use a computer keyboard and mouse to click and navigate by rotating, scaling and translating the rendered brain objects along the generated scene. Each registered population is visualized in SBA as a cloud of cubes that represents the 3D coordinates of 10 μm voxels containing labeled axons in RAS space (Left-Right, Posterior-Anterior, Inferior-Superior, with origin at the anterior commissure). To deal with the sparsity of labeled axons along the anterior-posterior axis due to the inter-spacing of coronal slices, a random shift is introduced along this axis to make the cubes look more spread out across the template. A pitfall of that approach is that the 10 μm resolution results in the point-cloud being converted to small 3D cubes which are hard to distinguish. To increase cube visibility, its linear size is multiplied by a factor of 20. For storing the data, the Extensible 3D (x3d) file format is used (see 1). Under the x3d format, the data are then uploaded to SBA via an Application Programming Interface.

1.6 Morphology selection

The MouseLight project/repository/database comprises of $\sim$ 1500 complete morphological reconstructions of single-neurons (Economo et al., 2016). These neurons are primarily located in the thalamus, motor cortex, subiculum and hypothalamus and their morphologies were reconstructed with a high-throughput procedure involving viral projection labeling, high resolution imaging and anatomical segmentation. They

first labeled single-neurons by injecting 56-days-old (P56) male wild-type mice with two Adeno-associated viruses (AAV): low-titer AAV expressing cre-recombinase (AAV Syn-iCre) and high-titer AAV coding for a green fluorescent protein (AAV CAG-Flex eGFP/tdTomato). To produce high-resolution images containing the labeled neurites, they used serial two-photon tomography with an integrated vibratome. For tracing and reconstructing the neurons, they implemented a semi-automated algorithm for the segmentation and reconstruction of the soma, axons and dendrites that required manual intervention for distinguishing branches at intersection points (Winnubst et al., 2019).

The Braintell project/repository/database comprises of $\sim$ 1700 fully reconstructed single-neuron morphologies from cortex, claustrum, thalamus, and striatum (Peng et al., 2021). This repository is a product of a pipeline designed for labeling, imaging, reconstructing, registering and analysing single-neurons from these areas. To label single-cells, they used a combination of two mouse transgenic lines, namely GFP-expressing Ai139 or Ai140 TIGRE2.0 reporter and the TIGRE-MORF reporter (Madisen et al., 2015; Daigle et al., 2018; Veldman et al., 2020), which lead to highly sparse labeling of the complete axonal and dendritic arborization of a cell. They thus performed anterograde tracing on $\sim$ 140 P56 male and female transgenic mice. They imaged whole-brains for each experiment using the fluorescence micro-optical sectioning tomography (fMOST) imaging platform (Li et al., 2010). fMOST is a platform combining epifluorescence microscope with a mechanical sectioning system. For each 3D imaged brain, they automatically reconstructed the underlying neuronal morphologies using the Vaa3D open source software (Peng et al., 2014), which were then registered to CCF with the mBrainAligner tool (Peng et al., 2011). They then used the above mentioned pipeline to characterize the morphological diversity of the aforementioned areas in terms of projection pattern-specific cell-types. These projection-specific types were found to be correlated with their transcriptomic composition, divergence/convergence of their projections, layer-specificity in the case of cortical neurons and somatotopic organization.

For integrating single-neuron morphological reconstructions in our analysis, we performed the following data retrieval and pre-processing steps. We first downloaded all VPM neurons from their respective repositories in the form of swc files (see 1 for the respective hyperlinks). The swc file format is the standardized format for representing morphological reconstructions (Stockley et al., 1993). We only selected morphologies whose soma position was located in the VPM. When selecting neurons from the Braintell database, we had to use a strict selection criterion based on the quality of the registration procedure. For each Braintell neuron, manual corrections were made to obtain its correct soma position to mitigate errors caused by their fully automated reconstruction pipeline. However, we only used neurons whose soma position was within the boundaries of VPM, and confirmed as VPM neurons by the authors in their supplementary tables after the manual corrections. Following this selection criterion, we thus retrieved 257 Mouselight and Braintell morphologies in total for analysis.

We loaded each morphology as a list of two arrays in which the first corresponded to the anatomical coordinate of each point in CCF and the second contained additional information about each point, such as its identity (soma, dendrite, axon), its index in

the first list and the index of its parent point in the list, where a parent point corresponded to its direct ancestor in the morphological tree that could be either an axonal branch, dendritic branch or the soma. We first had to ensure by means of an affine transformation that all neurons were represented in the same orientation system as CCF, which is PIR (Anterior-Posterior, Superior-Inferior, Left-Right with origin at the Anterior-Superior-Left corner) at a 10 μm resolution, which is the highest available resolution for the ARA template. Braintell neurons are oriented at PIR at 1 μm resolution, with only a small fraction having 25 μm resolution. We thus re-scaled the anatomical coordinates of the two Braintell groups by a factor of 10 and 0.4, respectively. Mouselight neurons are oriented at LIP (Right-Left, Superior-Inferior, Anterior-Posterior, corner origin) at 1 μm resolution. For the anatomical coordinates of each neuron, we switched the first and the third coordinate, mirrored the third coordinate and re-scaled all coordinates by a factor of 10.

Since we were exclusively interested in the axonal terminal branches, we first identified the terminals by finding axonal points that had no children. We then extracted the branch of a given terminal by backtracking from the terminal point to its ancestor points until a branching point was reached. We thereby obtained a list of the axonal terminal branches of each neuron across all somatosensory areas.

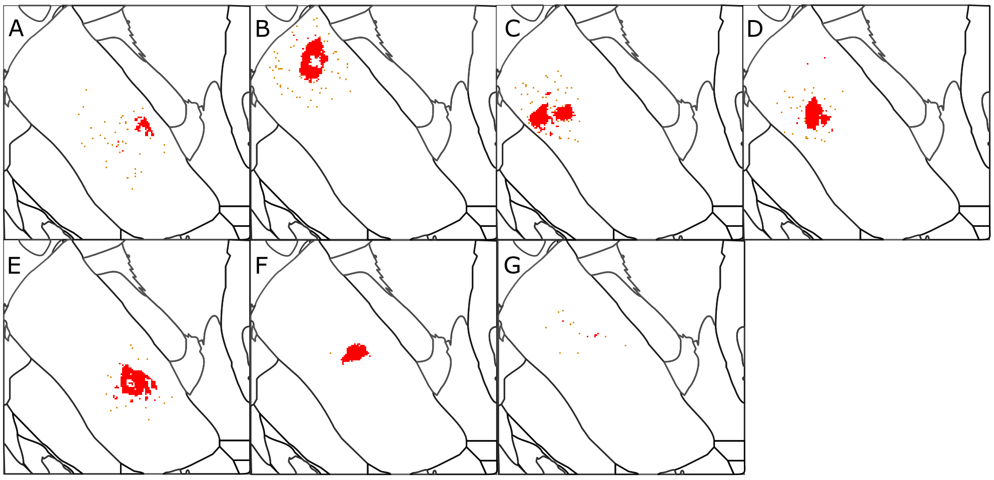

Fig. 1: Anatomical integration of registered population experiments with morphological reconstructions of long-range projecting single-neurons (LRPN) in the CCF suggests proximity between the two data modalities at the level of their source location inside VPM. (A-E) Maximum projection plots of VPM across the coronal plane (10 μm) that illustrate the proximity between the injection volume location of the populations and the soma location of LRPN neurons. The black contour lines delineate the anatomical borders of VPM according to the ARA parcellation (see section 2.6). The letters A to G are used to label the populations that correspond to the experiment ids 1-7. The red color represents the source location of a population and orange represents the soma location of proximal LRPN neurons.

References

Bakker, R., Tiesinga, P., and Kötter, R. (2015). The scalable brain atlas: Instant web-based access to public brain atlases and related content. *Neuroinformatics*, 13(3):353:366.

Berg, S., Kutra, D., Kroeger, T., Straehle, C. N., Kausler, B. X., Haubold, C., Schiegg, M., Ales, J., Beier, T., Rudy, M., Eren, K., Cervantes, J. I., Xu, B., Beuttenmueller, F., Wolny, A., Zhang, C., Koethe, U., Hamprecht, F. A., and Kreshuk, A. (2019). ilastik: interactive machine learning for (bio)image analysis. *Nature Methods*, 16(12):1226–1232.

Bishop, C. M. (2006). *Pattern Recognition and Machine Learning*. Information Science and Statistics. Springer, first edition.

Bowyer, A. (1981). Computing dirichlet tessellations*. *The Computer Journal*, 24(2):162–166.

Breiman, L. (2001). Random forests. *Machine Learning*, 45(1):5–32.

Carey, H., Pegios, M., Martin, L., Saleeba, C., Turner, A., Everett, N., Puchades, M., Bjaalie, J., and McMullan, S. (2022). Deepslice: rapid fully automatic registration of mouse brain imaging to a volumetric atlas. *bioRxiv*, page 2022.04.28.489953.

Chollet, F. (2017). Xception: Deep learning with depthwise separable convolutions. In *Proceedings of the IEEE Conference on Computer Vision and Pattern Recognition (CVPR)*.

Chon, U., Vanselow, D. J., Cheng, K. C., and Kim, Y. (2019). Enhanced and unified anatomical labeling for a common mouse brain atlas. *Nature Communications*, 10(1):5067.

Daigle, T. L., Madisen, L., Hage, T. A., Valley, M. T., Knoblich, U., Larsen, R. S., Takeno, M. M., Huang, L., Gu, H., Larsen, R., Mills, M., Bosma-Moody, A., Siverts, L. A., Walker, M., Graybuck, L. T., Yao, Z., Fong, O., Nguyen, T. N., Garren, E., Lenz, G. H., Chavarha, M., Pendergraft, J., Harrington, J., Hirokawa, K. E., Harris, J. A., Nicovich, P. R., McGraw, M. J., Ollerenshaw, D. R., Smith, K. A., Baker, C. A., Ting, J. T., Sunkin, S. M., Lecoq, J., Lin, M. Z., Boyden, E. S., Murphy, G. J., da Costa, N. M., Waters, J., Li, L., Tasic, B., and Zeng, H. (2018). A suite of transgenic driver and reporter mouse lines with enhanced brain-cell-type targeting and functionality. *Cell*, 174(2):465–480.e22.

Economo, M. N., Clack, N. G., Lavis, L. D., Gerfen, C. R., Svoboda, K., Myers, E. W., and Chandrashekar, J. (2016). A platform for brain-wide imaging and reconstruction of individual neurons. *eLife*, 5:e10566.

Griffiths, D. J. (2013). *Introduction to electrodynamics*. Pearson, Boston.

Groeneboom, N. E., Yates, S. C., Puchades, M. A., and Bjaalie, J. G. (2020). Nutil: A pre- and post-processing toolbox for histological rodent brain section images. *Frontiers in Neuroinformatics*, 14.

Harris, J. A. et al. (2018). The organization of intracortical connections by layer and cell class in the mouse brain. *bioRxiv*.

Harris, J. A. et al. (2019). Hierarchical organization of cortical and thalamic connectivity. *Nature*, 575:195–202.

Hintiryan, H., Foster, N. N., Bowman, I., Bay, M., Song, M. Y., Gou, L., Yamashita, S., Bienkowski, M. S., Zingg, B., Zhu, M., Yang, X. W., Shih, J. C., Toga, A. W.,

and Dong, H.-W. (2016). The mouse cortico-striatal projectome. *Nature Neuroscience*, 19(8):1100–1114.

Kim, Y., Yang, G. R., Pradhan, K., Venkataraju, K. U., Bota, M., García del Molino, L. C., Fitzgerald, G., Ram, K., He, M., Levine, J. M., Mitra, P., Huang, Z. J., Wang, X.-J., and Osten, P. (2017). Brain-wide maps reveal stereotyped cell-type-based cortical architecture and subcortical sexual dimorphism. *Cell*, 171(2):456–469.e22.

Li, A., Gong, H., Zhang, B., Wang, Q., Yan, C., Wu, J., Liu, Q., Zeng, S., and Luo, Q. (2010). Micro-optical sectioning tomography to obtain a high-resolution atlas of the mouse brain. *Science*, 330(6009):1404–1408.

Madisen, L., Garner, A. R., Shimaoka, D., Chuong, A. S., Klapoetke, N. C., Li, L., van der Bourg, A., Niino, Y., Egolf, L., Monetti, C., Gu, H., Mills, M., Cheng, A., Tasic, B., Nguyen, T. N., Sunkin, S. M., Benucci, A., Nagy, A., Miyawaki, A., Helmchen, F., Empson, R. M., Knöpfel, T., Boyden, E. S., Reid, R. C., Carandini, M., and Zeng, H. (2015). Transgenic mice for intersectional targeting of neural sensors and effectors with high specificity and performance. *Neuron*, 85(5):942–958.

Oh, S. W. et al. (2014). A mesoscale connectome of the mouse brain. *Nature*, 508:207–214.

Paxinos, G. and Franklin, K. B. J. (2019). *Paxinos and Franklin's The mouse brain in stereotaxic coordinates*. Academic Press, an imprint of Elsevier, London, fifth edition edition.

Peng, H., Bria, A., Zhou, Z., Iannello, G., and Long, F. (2014). Extensible visualization and analysis for multidimensional images using vaa3d. *Nature Protocols*, 9(1):193–208.

Peng, H., Chung, P., Long, F., Qu, L., Jenett, A., Seeds, A. M., Myers, E. W., and Simpson, J. H. (2011). Brainaligner: 3d registration atlases of drosophila brains. *Nature Methods*, 8(6):493–498.

Peng, H., Xie, P., Liu, L., Kuang, X., Wang, Y., Qu, L., Gong, H., Jiang, S., Li, A., Ruan, Z., Ding, L., Yao, Z., Chen, C., Chen, M., Daigle, T. L., Dalley, R., Ding, Z., Duan, Y., Feiner, A., He, P., Hill, C., Hirokawa, K. E., Hong, G., Huang, L., Kebede, S., Kuo, H.-C., Larsen, R., Lesnar, P., Li, L., Li, Q., Li, X., Li, Y., Li, Y., Liu, A., Lu, D., Mok, S., Ng, L., Nguyen, T. N., Ouyang, Q., Pan, J., Shen, E., Song, Y., Sunkin, S. M., Tasic, B., Veldman, M. B., Wakeman, W., Wan, W., Wang, P., Wang, Q., Wang, T., Wang, Y., Xiong, F., Xiong, W., Xu, W., Ye, M., Yin, L., Yu, Y., Yuan, J., Yuan, J., Yun, Z., Zeng, S., Zhang, S., Zhao, S., Zhao, Z., Zhou, Z., Huang, Z. J., Esposito, L., Hawrylycz, M. J., Sorensen, S. A., Yang, X. W., Zheng, Y., Gu, Z., Xie, W., Koch, C., Luo, Q., Harris, J. A., Wang, Y., and Zeng, H. (2021). Morphological diversity of single neurons in molecularly defined cell types. *Nature*, 598(7879):174–181.

Puchades, M. A., Csucs, G., Ledergerber, D., Leergaard, T. B., and Bjaalie, J. G. (2019). Spatial registration of serial microscopic brain images to three-dimensional reference atlases with the quicknii tool. *PLOS ONE*, 14(5):e0216796.

Stockley, E. W., Cole, H. M., Brown, A. D., and Wheal, H. V. (1993). A system for quantitative morphological measurement and electrotonic modelling of neurons: three-dimensional reconstruction. *Journal of Neuroscience Methods*, 47(1):39–51.

Tan, P. N., Steinbach, M., and Kumar, V. (2005). *Introduction to Data Mining*. Pearson, first edition.

Veldman, M. B., Park, C. S., Eyermann, C. M., Zhang, J. Y., Zuniga-Sanchez, E., Hirano, A. A., Daigle, T. L., Foster, N. N., Zhu, M., Langfelder, P., Lopez, I. A., Brecha, N. C., Zipursky, S. L., Zeng, H., Dong, H.-W., and Yang, X. W. (2020). Brainwide genetic sparse cell labeling to illuminate the morphology of neurons and glia with cre-dependent morf mice. *Neuron*, 108(1):111–127.e6.

Wang, Q., Ding, S.-L., Li, Y., Royall, J., Feng, D., Lesnar, P., Graddis, N., Naeemi, M., Facer, B., Ho, A., Dolbeare, T., Blanchard, B., Dee, N., Wakeman, W., Hirokawa, K. E., Szafer, A., Sunkin, S. M., Oh, S. W., Bernard, A., Phillips, J. W., Hawrylycz, M., Koch, C., Zeng, H., Harris, J. A., and Ng, L. (2020). The allen mouse brain common coordinate framework: A 3d reference atlas. *Cell*, 181(4):936 – 953.e20.

Watson, D. F. (1981). Computing the n-dimensional delaunay tessellation with application to voronoi polytopes*. *The Computer Journal*, 24(2):167–172.

Winnubst, J., Bas, E., Ferreira, T. A., Wu, Z., Economo, M. N., Edson, P., Arthur, B. J., Bruns, C., Rokicki, K., Schauder, D., Olbris, D. J., Murphy, S. D., Ackerman, D. G., Arshadi, C., Baldwin, P., Blake, R., Elsayed, A., Hasan, M., Ramirez, D., Santos, B. D., Weldon, M., Zafar, A., Dudman, J. T., Gerfen, C. R., Hantman, A. W., Korff, W., Sternson, S. M., Spruston, N., Svoboda, K., and Chandrashekar, J. (2019). Reconstruction of 1,000 projection neurons reveals new cell types and organization of long-range connectivity in the mouse brain. *Cell*, 179(1):268 – 281.e13.
